# Decoding Protein-Methylating METTLs in Humans: Structural, Functional, and Disease Insights over the Past Decade

**DOI:** 10.3390/ijms27146532

**Published:** 2026-07-22

**Authors:** Byron Baron

**Affiliations:** Centre for Molecular Medicine and Biobanking, University of Malta, MSD2080 Msida, Malta; byron.baron@um.edu.mt

**Keywords:** methyltransferase-like (METTL) enzymes, protein methyltransferases, colorectal cancer, glioblastoma

## Abstract

Methylation of proteins is a critical post-translational modification that regulates diverse cellular processes, including signal transduction, protein stability, and enzymatic activity. The methyltransferase enzymes that catalyse the addition of such methyl groups onto target molecules fall into a wide variety of categories and as such are classified into numerous families. Among them, the methyltransferase-like (METTL) family represents a unique cluster of enzymes with structural similarity to arginine methyltransferases. This family comprises 27 members, many of which methylate lysine residues on proteins, while others target various forms of RNA. Although discovered just over a decade ago, the protein-methylating METTLs remain incompletely characterised. Notably, most identified protein substrates are non-histone proteins, underscoring the distinctive functional roles of these enzymes. This review focuses exclusively on the protein-methylating METTL family members, summarising current knowledge of their structural features, enzymatic targets, sub-cellular localisation, and expression patterns. Their emerging relevance to disease, particularly cancer, is also highlighted, alongside areas where mechanistic understanding remains limited. By consolidating recent advances, this review aims to provide a comprehensive overview of protein-methylating METTLs in humans and to identify the critical knowledge gaps that will guide future research into their biological roles and therapeutic potential.

## 1. Introduction

Methyltransferases are enzymes that catalyse the transfer of a methyl group (CH_3_) from a donor, generally S-adenosyl-L-methionine (SAM or AdoMet), to a wide range of substrate molecules covering proteins, DNA, RNA, lipids, as well as small biomolecules including metabolites [1,2,3].

**Figure 1:**
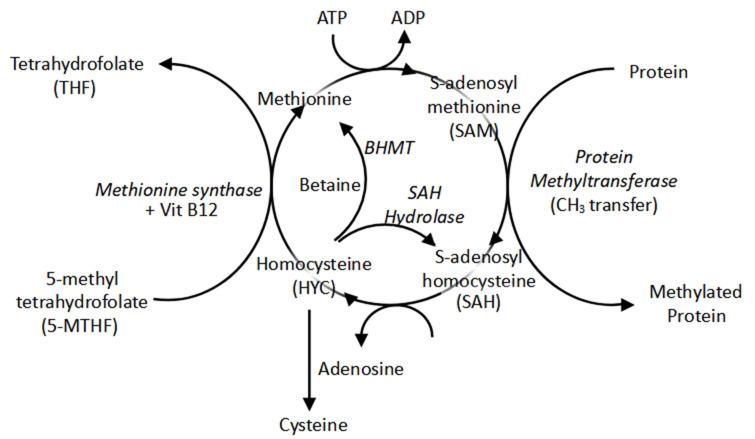
Overview of the methionine cycle and S-adenosylmethionine (SAM)-dependent protein methylation. Methionine is converted into SAM, the universal methyl donor for protein methyltransferases, including members of the METTL family. Following methyl group transfer to protein substrates, S-adenosylhomocysteine (SAH) is generated and subsequently recycled through the methionine cycle (modified from [7]).

This is made possible through the use of a methyl donor, which in most cases is s-adenosylmethionine (SAM), that for this reason is considered a universal methyl donor. SAM is synthesised from the conjugation of adenosine produced from the hydrolysis of adenosine triphosphate (ATP) with methionine in a reaction catalysed by SAM synthetase (a.k.a., methionine adenosyltransferase; MAT) [4]. The addition of a methyl group results in the formation of S-adenosylhomocysteine (SAH or AdoHC) as a by-product, which in turn is hydrolysed by adenosylhomocysteinase (AHCY) to homocysteine (Hcy). Homocysteine is then re-methylated to regenerate methionine using 5-methyltetrahydrafolate (5-MTHF) or betaine via betaine homocysteine methyltransferase (BHMT) [5]. This sequence of reactions form the basis of the methyl cycle [6] (Figure 1).

Besides being categorised based on the substrates they methylate, SAM-dependent methyltransferases can also be classified based on their structural and biochemical features, sub-dividing them into five distinct classes of enzymes. Class I enzymes are characterised by a seven-strand twisted β-sheet (a Rossmann fold motif). Class II enzymes are characterised by a long, central, antiparallel b-sheet flanked by groups of helices at either end. Class III enzymes are characterised by an active site buried within a cleft between two αβα domains, each containing five strands and four helices. Class IV enzymes—called SPOUT (SpoU-TrmD) enzymes for the founding members of the class—SpoU (renamed TrmH) and TrmD are characterised by a six-stranded parallel β-sheet flanked by seven α-helices, of which the first three strands form a deep trefoil knot in the structure, i.e., half a Rossman fold. Class V enzymes are characterised by a series of eight curved β-strands forming three sheets, with a fold organised around a pseudo-knot called the Su (var), E (z) and Trithorax (SET) domain [3].

Despite the differences that methyltransferases present across the five classes, and irrespective of which type of substrate they catalyse, they all share a catalytic domain comprising a SAM-binding pocket, that is very tightly conserved within each class, adjacent to a substrate-acceptor pocket which is highly variable in order to provide substrate specificity [8,9]. Linking the two pockets is a hydrophobic channel that enables the transfer of the CH_3_ group found on SAM donor onto the target molecule via an S_N_2 transition [10].

### Protein Methylation

Protein methylation is one of the more widespread post-translational modifications. The addition of one or more methyl groups can alter numerous features of proteins, notably sub-cellular localisation, protein half-life (stability/turn-over), activity, and molecular interactions (with target proteins or other molecules) [11,12].

Methylation can be added onto nitrogen, oxygen, or sulfur atoms. N-methylation is most frequently found on the side chains of lysine and arginine, although histidine, alanine, proline, glutamine, phenylalanine, asparagine, and methionine residues can also undergo such additions [13]. O-methylation is added to aspartic acid, glutamic acid and cysteine residues, and S-methylation is added to methionine and cysteine residues [14]. Additionally, methylation can be added to protein N- and C-termini [15].

The protein methyltransferases are grouped into families based on their distinct structural domains, with all protein arginine methyltransferases (PRMTs) belonging to Class I (seven-β-strand methyltransferases), while most protein lysine methyltransferases (PKMTs) belong to Class V (SET domain methyltransferases) [2,3]. Other PKMTs fall within Class I as in the case of disruptor of telomeric silencing 1-Like (DOT1L) and methyltransferase-like (METTL) family members [16]. PRMTs catalyse mono-(Rme1), asymmetric di-(aRme2) or symmetric di-(sRme2) methylation on the guanidinium moiety of arginine residues, while PKMTs catalyse mono-(Kme1), di-(Kme2) or tri-(Kme3) methylation on the epsilon (ε) amine of lysine residues [17].

Within this broader enzymatic landscape, the METTL family represents a particularly intriguing group of protein methyltransferases, distinguished by their structural similarity to arginine methyltransferases and their largely non-histone protein substrates. The following section provides an overview of the METTL family members, with emphasis on those that target proteins.

## 2. The METTL Family Members

**Figure 2:**
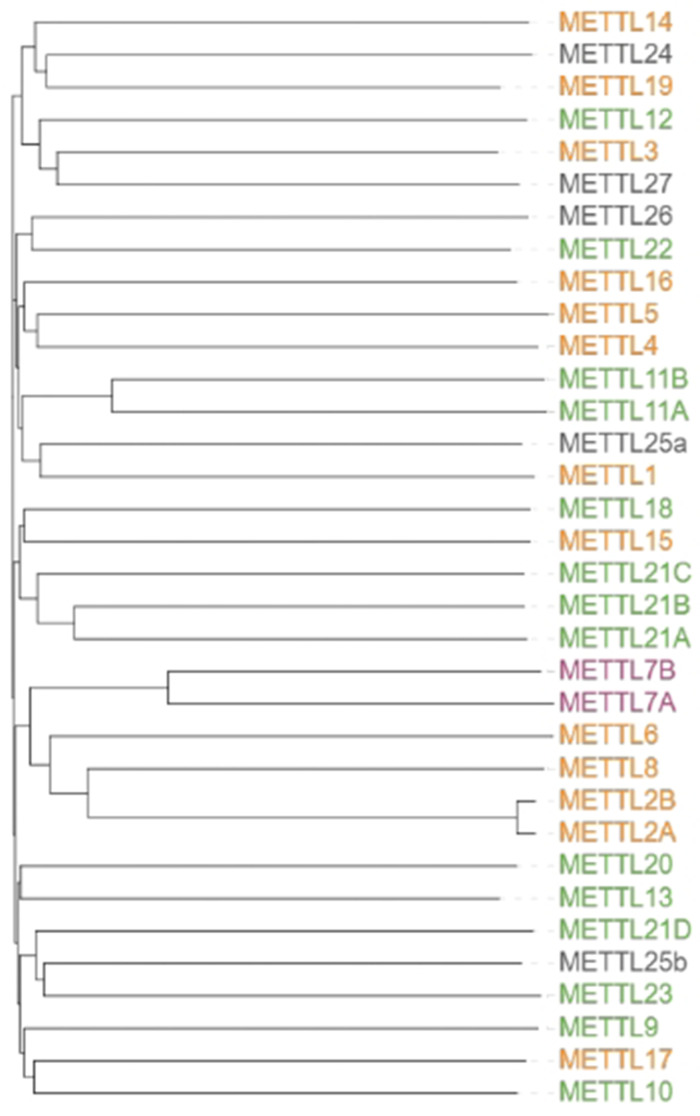
Human METTL proteins phylogeny tree. Colour legend: orange—RNA methyltransferase; green—protein methyltransferase; purple—other biomolecules; grey—unknown substrate (sequences retrieved from Uniprot, aligned using T-Coffee and tree built using iTOL [24]).

The METTL family is an artificial grouping of proteins with a putative methyltransferase function based solely on sequence homology. In fact, they present significant diversity in terms of primary amino acid sequence. Thus, while all METTLs share some conserved features, the breadth of the diversity within the family necessitates a practical sub-classification of the members in order to generate meaningful generalisations. The simplest form of diversification is based on the type of substrate molecule targeted by each enzyme, which separates most members into one group acting on nucleic acids (mainly RNAs) and another group that methylate proteins (Table 1), however structurally they are not so easily segregated (Figure 2).

One of the earliest systematic identification of a sub-group of the METTL family members via sequence homology involved using local alignment of METTL22, which retrieved a group of 10 distantly related proteins suspected of being seven-β-strand (Class I) methyltransferases, namely METTL18, METTL20, METTL21A, METTL21B, METTL21C, METTL21D, METTL22, METTL23, Calmodulin-Lysine N-Methyltransferase (CAMKMT) and Eukaryotic Elongation Factor 2 Lysine Methyltransferase (EEF2KMT; FAM86) [19]. These proteins were at the time considered putative methyltransferases clustered within the so-called “Group J” (2). Since then, these enzymes have been established as bona fide protein methyltransferases, although their precise biological functions, substrate specificities, and regulatory mechanisms remain incompletely characterised.

While RNA-targeting METTLs such as METTL3 [20,21] and METTL16 [22,23] have been extensively characterised in the context of epitranscriptomic regulation, the protein-methylating subgroup remains comparatively understudied. In the following sections, the focus will be exclusively on the protein-methylating METTL enzymes, reviewing their structural features, enzymatic targets, biological functions, and emerging relevance to disease.

## 3. METTL Enzyme Structural Homology

**Figure 3:**
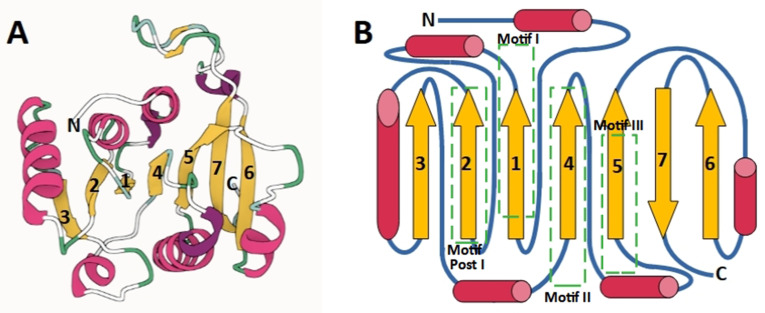
General structure of METTL proteins. (**A**) The predicted structure of METTL23 (AlphaFold prediction: AF-Q86XA0-F1; 190 amino acids) visualised using Mol* Viewer 5.0.0. (**B**) A cartoon representation of METTL23 (Q86XA0). Colour legend: yellow—β-strands; pink—α-helices. (Based on [16]).

Like all other seven-β-strand methyltransferases, the core structure of the METTL family members consists of six parallel β-strands, and a seventh strand with the reverse orientation, alternating with six α-helices, that flank the β-sheet (three on either side). Such enzymes have the four characteristic conserved sequence motifs (Motif I, Post I, Motif II, and Motif III) characteristic of this class (Figure 3). The SAM-binding region is located at the N-terminal side of the β-sheet, with the conserved residues found in Motif I and Post I being essential for the docking of the SAM methyl donor [25]. The substrate-binding region is located at the C-terminal side of the β-sheet and since the targets methylated by these enzymes are very diverse in terms of 3D shape, size and chemistry, the substrate-binding region presents a similarly broad variation in structure and topology between the METTL family members [1,3].

Similar to other seven-β-strand methyltransferases, there is an indication for METTL family members to recognise the folded three-dimensional structural features of specific regions within their substrates, rather than a linear peptide sequence [16]. This has been demonstrated through the interaction of METTL21D with its target methylation site lysine 315 (K315) on Valosin-containing protein (VCP; a.k.a., transitional endoplasmic reticulum ATPase or p97), whereby the mutation of residues flanking K315 did not prevent the methylation from being added [26]. This indicates the recognition of the folded protein surface rather than the sequence context and a similar principle is likely to apply across all other protein-methylating METTLs.

Comparing sequences and secondary structures confirms that whilst the METTL enzymes retain their conserved catalytic features, they vary in overall size and inclusion of additional domains which further diversify the substrate-binding surfaces, reflecting adaptation towards a highly diverse set of protein targets [27].

Taken together these findings show that while the protein-methylating METTLs share a conserved catalytic core, they display substantial divergence in those regions that form substrate-binding surfaces. This variation underpins their ability to target such a broad range of structurally distinct protein substrates.

## 4. METTL Protein Methyltransferase Targets and Localisation

Based on data gathered so far, it is clear that protein methyltransferases of the METTL family act on a diverse but highly specific set of protein substrates (Table 2). A striking feature is that each of these METTL enzymes appears to methylate either a single protein or else a group of closely related proteins (e.g., the HSP70 family by METTL21A). However, there is no overarching functional theme linking the various substrates. Instead, each modification has a distinct biochemical consequence, when such roles have been elucidated. It is thus interesting how this group of methyltransferases, despite presenting limited biological significance (hence their late discovery), have evolved to play such a unique role in cellular processes.

Consequently, sub-cellular localisation patterns reflect this substrate diversity (Table 3). METTL proteins are found in multiple compartments, including mitochondria, nucleus, cytoplasm, and endoplasmic reticulum, in order to match the functional distribution of their protein targets. For example, mitochondrial METTL12 methylates citrate synthase, while cytoplasmic METTL21A acts on HSP70 chaperones. Such localisation reinforces the lack of a unifying functional role across the family, with individual enzymes adapting to niche cellular contexts.

Kinetic studies indicate that METTL enzymes act in a distributive fashion, i.e., following each methyl transfer event, the enzyme dissociates from the substrate before rebinding with a new SAM donor. Each methyl group added changes the substrate slightly and, in some cases, the affinity of the methyltransferase for the substrate or the positioning of the substrate in the active site can be substantially altered, thereby influencing the potential for further methylation [16]. This distributive mechanism permits substrates to exist in multiple methylation states simultaneously. This has been shown in vitro for METTL 21D, which generated a mixture of mono-, di- and trimethylation on VCP [26], and has been proposed for METTL20, METTL21A and EEF2KMT through their action on ETFβ, HSPA1 and eEF2, respectively, which presented a mixture of methylation states in vivo [37,38]. Although the exact stoichiometry can vary, and at any one time a target protein could be present in multiple methylation states, it is likely that all lysine methyltransferases within the METTL family are capable of achieving full trimethylation of their targets (Table 4).

An important consideration is the biological significance, and consequently the evolutionary origin of these unique protein methyltransferases. As mentioned previously, the METTL family is an artificial grouping united by structural homology rather than common ancestry. The marked sequence diversity observed in the primary sequences of METTL family enzymes protein-methylating METTLs supports the notion of parallel or convergent evolution, whereby distinct enzymes independently acquired methyltransferase activity, as opposed to them originating from a common ancestor [16]. This has implications on the reason behind their existence in nature and the role they play in biochemistry, raising the possibility that METTLs represent an evolutionary solution to highly specific cellular demands, rather than originating from the diversification within an enzymatic family with shared biological roles [44].

## 5. METTL Protein Methyltransferase RNA and Protein Expression Levels

The RNA and protein expression levels in various body tissues (Table 5) offer a potential indication of which organs these enzymes perform their function most prolifically in and what is the significance of their roles in cellular biology. However while RNA and protein expression patterns can hint at relevant locations for further investigation, the mismatch between transcript levels and protein abundance complicates interpretation. Furthermore, high protein expression does not necessarily imply high enzyme activity.

For example, as shown in Table 5, several METTLs show high RNA enrichment in spermatids (METTL11A, METTL13, METTL22, METTL23), suggesting potential roles in germ cell differentiation, yet protein evidence is either sparse or highly variable across tissues. Conversely, METTL12 exhibits moderate RNA expression in alveolar and bronchial cells but relatively strong protein expression in renal and reproductive tissues, highlighting the importance of post-transcriptional and post-translational regulation. Interestingly, renal tubules emerge as a recurrent site of high RNA or protein abundance (METTL9, METTL10, METTL12, METTL18, METTL20), hinting at a broader involvement of METTL-mediated methylation in renal physiology or mitochondrial function within this tissue. Likewise, immune and epithelial cell types show isolated enrichments (e.g., monocytes for METTL21B, keratinocytes for METTL21D, small intestinal crypts for METTL21A), suggesting highly specialised, context-dependent roles.

Overall, the expression data underscore the diversity of the protein-methylating METTLs and reinforce the idea that their biological functions are not unified by substrate type or cellular pathway. Instead, each enzyme appears to have evolved highly specific expression niches, the biological rationale of which remains poorly understood.

## 6. Relevance to Disease

Whilst biochemical characterisation of some protein-methylating METTL enzymes has accelerated in recent years, the family remains unevenly studied. Enzymes such as METTL9, METTL11A, METTL18 and METTL21A have been mechanistically investigated but many other members remain defined primarily through substrate identification or initial functional studies, highlighting significant opportunities for future research. Consequently, publicly available multi-omic resources provide a valuable complementary approach for systematically comparing the protein-methylating METTL family across diverse biological contexts, identifying patterns of genomic, transcriptomic and proteomic dysregulation, and prioritising candidates for future mechanistic investigation.

The protein-methylating METTLs have been implicated in the development and progression of various human diseases, spanning cancers, neurological disorders and multisystemic conditions. Although associations with multiple disease areas have been reported, the literature is heavily skewed toward cancer, where dysregulation of several METTL enzymes is increasingly recognised as contributing to tumour biology and progression. For example, METTL11A has been associated with AML prognosis [46], while METTL13 promotes tumorigenesis in lung and pancreatic cancer [47], METTL18 regulates translation in cancer cells [36] and METTL21A has been implicated in several human cancers, including lung cancer [48] and hepatocellular carcinoma [49].

Although considerably less studied than cancer, emerging evidence suggests that protein-methylating METTL dysfunction can contribute to neurological phenotypes. Mutations in METTL23 have been linked to autosomal recessive intellectual disability and impaired transcriptional regulation [50,51]. Similarly, METTL21C and METTL21D have been loosely associated with Alzheimer’s disease, although direct evidence is lacking [52]. METTL12, through its activity on citrate synthase, links directly to mitochondrial metabolism, and its dysregulation could influence metabolic homeostasis [34]. METTL9 has also been shown to play a role in sustaining neurogenesis and neural development through maintenance of the secretory system particularly related to the Golgi [53] and this could have implications in neurological diseases. Interestingly, METTL9 methylation of SLC39A7 has been implicated in osteoporosis through reduced bone loss in an ovariectomy mouse model [54]. Collectively, these observations indicate that even subtle perturbations in methyltransferase activity can disrupt neuronal pathways and this highlights the potential importance of METTL enzymes in neuronal, metabolic and multisystemic pathologies, although the underlying mechanisms remain poorly understood.

To complement the published functional studies discussed above, the remainder of this section integrates analyses of publicly available multi-omic resources to provide a systematic comparison of the protein-methylating METTL family across human cancers. Given the limited disease-specific literature available for many family members, these datasets provide valuable insights into patterns of genomic alteration, transcript abundance, protein expression and gene dependency that can help prioritise future mechanistic investigation. Throughout this section, observational findings derived from public datasets are interpreted as associations and hypothesis-generating evidence rather than proof of causality.

### Relevance to Cancer

To complement the published literature and better understand the biological role of the protein methyltransferase-like (METTL) family in cancer biology, publicly available multi-omic cancer resources were analysed to systematically compare the protein-methylating METTL family across diverse tumour types. An initial analysis was performed using the comprehensive cell line data available through the Cancer Cell Line Encyclopaedia (CCLE) repository, a comprehensive public resource containing genomic, transcriptomic and proteomic data from more than 1000 extensively characterised human cancer cell lines ([55,56] CCLE was accessed on 21 July 2025 from https://registry.opendata.aws/ccle). Cell lines were selected as a starting point given the relative homogeneity of their cell populations, the reproducibility they afford across independent laboratories, and their widespread use in exploratory and characterisation studies, thus facilitating comparative analyses across tumour types for identifying broad biological patterns.

**Figure 4:**
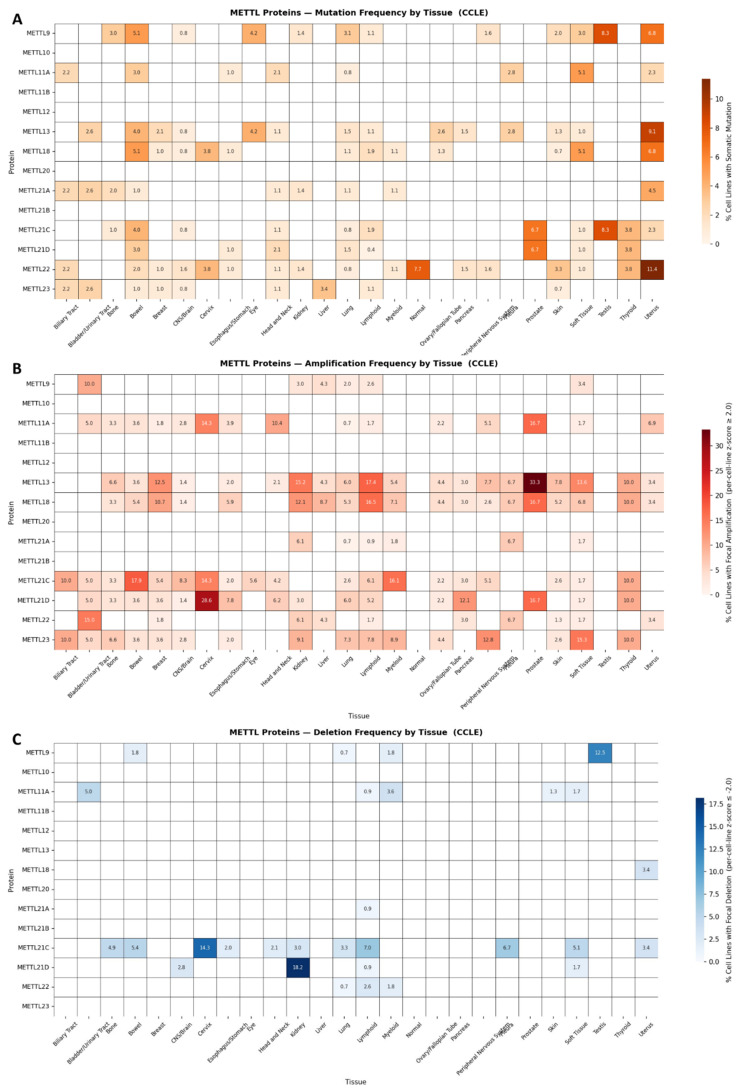
Genomic alteration frequency for the METTL family protein methyltransferases across CCLE cell lines, grouped by tissue. (**A**) Somatic mutation frequency (% of cell lines harbouring a somatic mutation) per METTL gene and tissue. (**B**) Focal amplification frequency (% of cell lines with a per-cell-line copy number z-score ≥ 2.0) per METTL gene and tissue. Colour intensity in each panel is proportional to frequency; blank cells indicate no alteration was detected in that tissue for the given protein. (**C**) Focal deletion frequency (% of cell lines with a per-cell-line copy number z-score ≤ −2.0) per METTL gene and tissue.

Genomic profiling was first performed using copy number (whole-genome sequencing-derived) and somatic mutation data from CCLE. An overview of mutation frequency per tissue was generated, flagging tissues in which mutation frequency for a given METTL gene exceeded a 5% enrichment threshold (Figure 4A and Figure S1). This analysis identified a small set of tissue-specific enrichments: METTL9 and METTL18 in bowel cancer, METTL21C and METTL21D in prostate cancer, METTL11A and METTL18 in soft tissue cancers, METTL9 and METTL21C in testicular cancer, and METTL9, METTL13, METTL18 and METTL22 in uterine cancer. Subsequent analysis of focal amplifications and focal deletions (Figure 4B,C) showed that focal amplification was widespread, most notably affecting METTL13, METTL18, METTL21C, METTL21D and METTL23. With these five amplified genes distributed across different chromosomes, this ruled out a single recurrent chromosomal event as the underlying cause. Focal deletion, by contrast, was comparatively rare overall, occurring most recurrently in METTL21C (cervix, lymphoid and peripheral nervous system cancers) and most prominently in METTL21D (kidney, ~18.2%).

**Figure 5:**
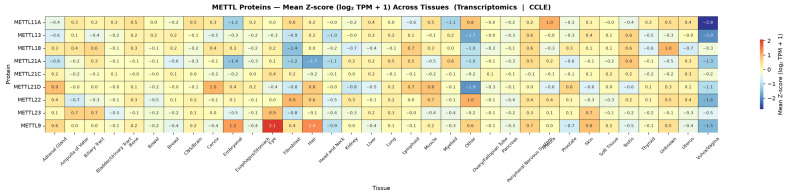
Transcriptomic profiling of the METTL family protein methyltransferases across CCLE cell lines, grouped by tissue. Mean z-score of log_2_(TPM + 1) RNA expression per METTL gene and tissue. Red shading denotes elevated expression and blue shading denotes reduced expression relative to the pan-tissue mean, with colour intensity proportional to magnitude.

Transcriptomic profiling was subsequently performed using the Cancer Dependency Map (DepMap) 26Q1 RNA-seq expression data [57]. DepMap is a public resource integrating molecular profiling and functional genomic data across a large panel of human cancer cell lines. Mean transcript abundance (log_2_(TPM + 1)) was summarised for human protein-coding genes, with mean z-scores calculated across cell lines within each tissue (Figure 5 and Figure S2). Overall, transcript abundance varied relatively little across the protein METTLs, with most members remaining close to the pan-cancer average across tissues. The most pronounced variation was observed for METTL9, with elevated expression in ocular (+2.1), hair (+1.4) and embryonal tissues (+1.2), and reduced expression in vulva/vagina (−1.5). METTL11A also showed notable tissue-specific variation, with elevated expression in prostate (+1.0) and reduced expression in vulva/vagina (−2.8), embryonal (−1.2) and myeloid (−1.1) tissues. Smaller elevations were noted for METTL21D (adrenal, cervix, lymphoid and muscle tissues) and METTL22 (fibroblast).

**Figure 6:**
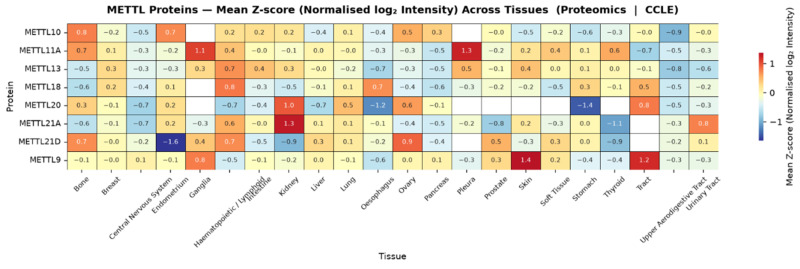
Proteomic profiling of the METTL family protein methyltransferases across CCLE cell lines, grouped by tissue. Mean z-score of normalised log_2_ protein intensity (TMT) per METTL gene and tissue. Red shading denotes elevated expression and blue shading denotes reduced expression relative to the pan-tissue mean, with colour intensity proportional to magnitude. Blank cells indicate the protein was not detected in that tissue.

To determine whether transcript abundance was reflected at the protein level, protein expression was investigated using CCLE consortium, TMT-quantified, normalised proteomic data (Figure 6 and Figure S2). Protein abundance exhibited considerably greater tissue-specific heterogeneity than the transcript abundance, despite incomplete proteomic coverage (METTL21C, METTL22 and METTL23 were not detected at the protein level in this dataset). The most notable increases in abundance were observed for METTL9 in skin (+1.4), METTL11A in pleura (+1.3), METTL21A in kidney (+1.3) and METTL20 in kidney (+1.0). Both METTL21A and METTL20 showed a contrasting reduction elsewhere, i.e., METTL21A in thyroid (−1.1) and METTL20 in stomach (−1.4) and oesophagus (−1.2). A further notable decrease was observed for METTL21D in endometrium (−1.6), despite a modest increase in ovary (+0.9).

Collectively, these transcriptomic and proteomic analyses indicate that while most protein-methylating METTLs exhibit relatively stable expression across cancer types, several family members display marked tissue-specific expression patterns that may reflect specialised biological functions. These observations provide a framework for prioritising future functional studies but should not be interpreted as evidence of disease causality.

**Figure 7:**
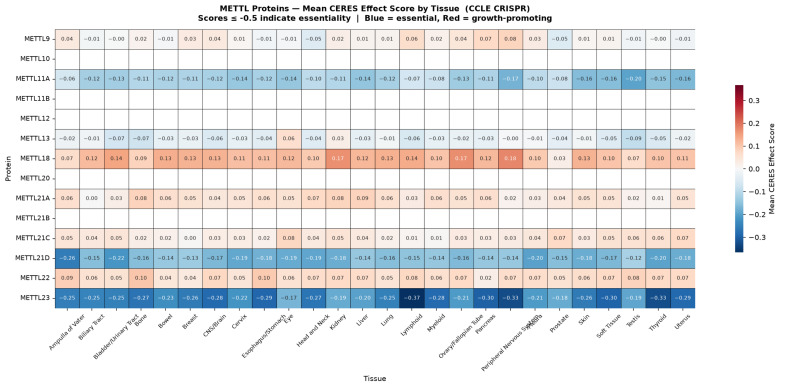
Mean CERES dependency effect scores for the METTL family protein methyltransferases across 26 tissue types in CCLE cancer cell lines. Each cell shows the tissue-averaged CERES score derived from DepMap CRISPR knockout screens; scores ≤ −0.5 indicate gene essentiality. Blue shading denotes negative (growth-supporting) effect scores, red shading denotes positive (growth-promoting) scores, and colour intensity is proportional to magnitude. Blank rows indicate proteins not screened in this dataset.

To further explore the functional relevance of the protein-methylating METTLs, CERES gene effect and dependency scores derived from DepMap genome-wide CRISPR knockout screens were examined (Figure 7 and Figure S3). CERES scores estimate the impact of gene loss on cellular fitness, with increasingly negative values indicating greater dependency [58]. Although no METTL protein reached the conventional essentiality threshold (CERES ≤ −0.5), three members showed a consistent negative trend across nearly all tissue types. METTL23 was the most prominent (peaking at −0.37 in lymphoid and −0.33 in peripheral nervous system and thyroid cancers), whilst smaller but similarly broad signals were obtained for METTL21D and METTL11A. In contrast, METTL18 displayed a consistent positive CERES score across almost all tissues (peaking at +0.17–0.18 in kidney, ovary/fallopian tube and peripheral nervous system), with a smaller equivalent pattern for METTL22, potentially indicating that these proteins are more compatible with growth-promoting rather than growth-supporting roles for these two family members under the conditions represented in these cell line models. METTL9, METTL13, METTL21A and METTL21C showed no consistent dependency signal in either direction, and CERES data were unavailable for METTL10, METTL11B, METTL12, METTL20 and METTL21B.

Although none of the protein-methylating METTLs fulfilled the conventional threshold for pan-cancer essentiality, the consistent tissue-independent trends observed for METTL11A, METTL18, METTL21D, METTL22 and METTL23 identify these enzymes as candidates for future functional investigation. As with the preceding transcriptomic and proteomic analyses, these findings represent associations derived from public functional genomic datasets and should be interpreted as hypothesis-generating rather than evidence of causal roles in tumour biology.

**Figure 8:**
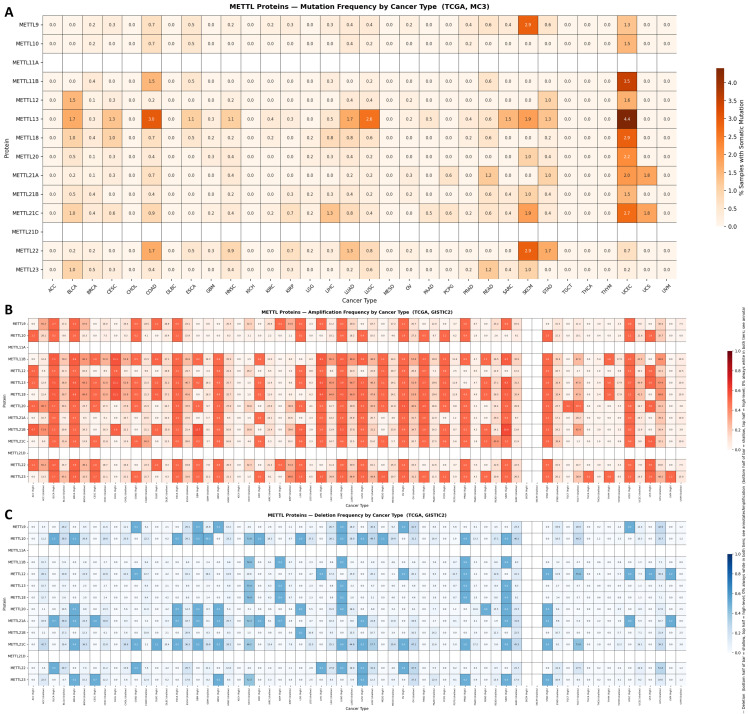
Genomic alteration frequency for the METTL family protein methyltransferases across TCGA samples, grouped by cancer type. (**A**) Somatic mutation frequency (% of samples) for the METTL family protein methyltransferases across 33 TCGA cancer types (Multi-Centre Mutation Calling in Multiple Cancers (MC3) project mutation calls). Each cell shows the percentage of tumour samples within a given cancer type harbouring a somatic mutation in the corresponding METTL gene; colour intensity is proportional to mutation frequency. Blank rows indicate proteins not assessed in this dataset. (**B**) Focal and broad copy number amplification frequency (%) for the METTL family protein methyltransferases across TCGA cancer types (GISTIC2). (**C**) Focal and broad copy number deletion frequency (%) for the METTL family protein methyltransferases across TCGA cancer types (GISTIC2). Each cancer type is divided into “high” (focal, deep deletion) and “shallow” (broad, arm- or chromosome-level deletion) sub-columns; colour intensity is proportional to the percentage of samples affected within each tier. Blank rows or columns indicate proteins or cancer types not assessed.

Following this initial screening in cell lines, genomic alterations were investigated in primary human tumours using The Cancer Genome Atlas (TCGA dataset https://www.cancer.gov/ccg/research/genome-sequencing/tcga; last accessed on 7 June 2026), a comprehensive public resource containing genomic, transcriptomic and clinical data from over 10,000 patients representing 33 cancer types [59]. Somatic mutations and copy number alteration data were examined per tissue, applying the same 5% enrichment threshold as the CCLE analysis (Figure 8A). This identified METTL13 as the most frequently mutated METTL family member, both in breadth (with non-zero mutation frequency recorded across the majority of tumour types) and in magnitude, reaching the highest single value in the panel (4.4% in UCEC). Uterine corpus endometrial carcinoma (UCEC) showed the greatest number of mutated METTLs of any tumour type, with elevated frequencies recorded across nearly the entire family. Analysis of focal (high-level) and broad (shallow) copy number alteration (Figure 8B,C) showed that, in contrast to the comparatively sparse CCLE cell line data, both deletion and amplification events were widespread across the TCGA tumour panel. Notably, the largest-magnitude changes were frequently observed in the shallow rather than the focal tier, indicating that many of the observed alterations likely reflect broader chromosome- or arm-level copy number events rather than gene-specific selection.

**Figure 9:**
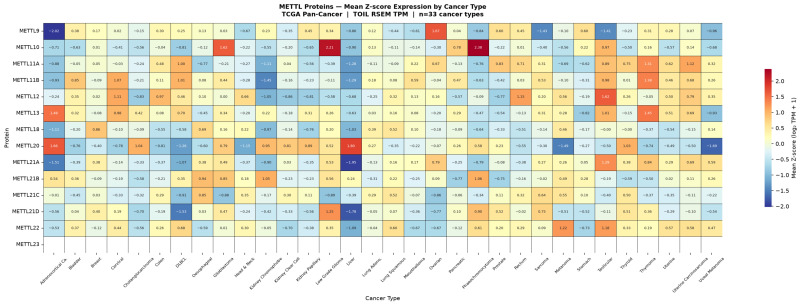
Transcriptomic profiling of the METTL family protein methyltransferases across 33 TCGA pan-cancer tumour types. Mean expression z-score for the METTL family protein methyltransferases across 33 TCGA pan-cancer tumour types (TOIL RSEM, log_2_(TPM + 1)). Each cell shows the tissue-averaged z-score relative to the pan-cancer mean; red shading denotes elevated expression and blue shading denotes reduced expression, with colour intensity proportional to magnitude. A blank row indicates a protein not detected in this dataset.

The transcript abundance was similarly interrogated across the TCGA pan-cancer panel using TOIL-processed RNA sequencing data (RSEM TPM, *n* = 33 cancer types) (Figure 9 and Figure S4). Primary tumours displayed substantially greater tissue-specific variation in METTL transcript abundance than was observed in the CCLE cell line dataset. Liver tissue emerged as a notable hotspot of extreme values across multiple family members, albeit in divergent directions: METTL10 (+2.38) and METTL20 (+1.80) showed their strongest pan-cancer elevation in liver, while METTL11A (−1.28), METTL11B (−1.29), METTL21A (−1.95) and METTL21D (−1.78) showed their strongest reduction in the same tissue. Notably, METTL23 was not detected in this tumour transcriptomic dataset, contrasting with the dependency signal observed in the DepMap CRISPR screens. This raises questions as to whether the CRISPR dependency signal observed for this protein in cell lines reflects a biologically meaningful difference between cancer cell lines and primary tumours, tumour heterogeneity, or technical aspects of the respective datasets and warrants further investigation.

Overall, the greater tissue-specific variability observed in the TCGA transcriptomic analyses compared to cancer cell lines, emphasises the importance of validating observations across complementary model systems. These expression patterns provide a useful framework for prioritising future functional studies but should not be interpreted as evidence of causal roles in tumour development.

**Figure 10:**
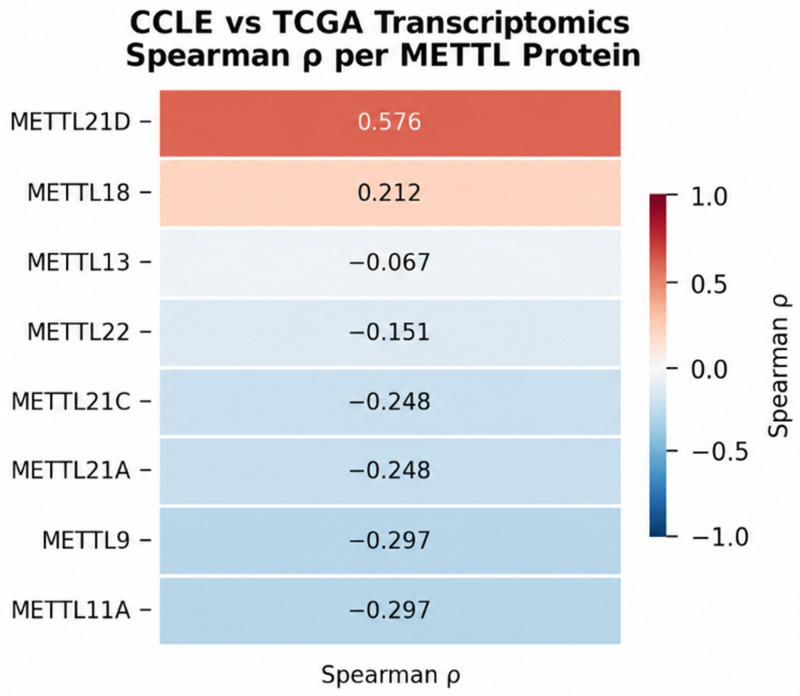
Spearman correlation coefficients (ρ) between CCLE cell line and TCGA tumour mean expression z-scores, shown for the subset of METTL family protein methyltransferases detected transcriptomically in both datasets. Red shading denotes positive correlation and blue shading denotes negative (inverse) correlation, with colour intensity proportional to the magnitude of ρ.

Given the apparent differences in transcriptomic patterns between the CCLE and TCGA datasets, a Spearman correlation analysis was performed between cell line and tumour expression z-scores for the eight METTL proteins detected in both datasets. This restricted set reflects a coverage asymmetry between the two platforms, where TCGA transcriptomic profiling detected the large majority of the METTL family (with only METTL23 absent), whereas CCLE transcriptomic coverage extended to only 9 of the 14 family members, limiting direct comparison to those proteins quantified in both datasets (Figure 10 and Figure S5). Of this overlapping set, only METTL21D showed a correlation of meaningful positive magnitude (ρ = 0.576), with METTL18 showing a weaker positive relationship (ρ = 0.212). METTL13 and METTL22 showed negligible correlation (ρ = −0.067 and −0.151 respectively), while METTL21C, METTL21A, METTL9 and METTL11A displayed a consistent inverse relationship between cell line and tumour expression (ρ = −0.248 to −0.297). Taken together, these findings indicate that for the majority of METTL family members assessable by this comparison, expression patterns observed in CCLE cell lines do not reliably predict, and in several cases directly contradict, those observed in primary TCGA tumours, underscoring the risk of drawing biological inferences about METTL regulation in cancer from cell line models in isolation.

**Figure 11:**
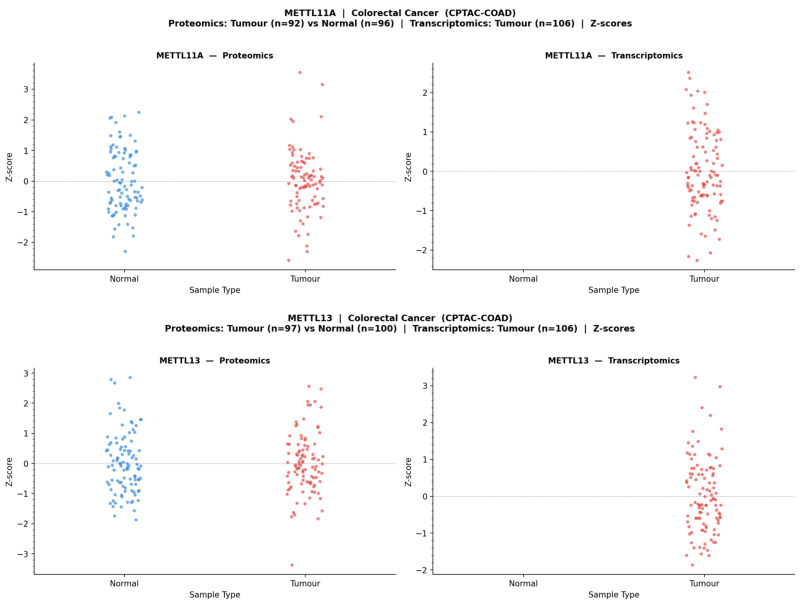
Proteomic and transcriptomic expression of METTL11A and METTL13 in colon adenocarcinoma (COAD) from the CPTAC dataset. Proteomic coverage was only observed for METTL11A and METTL13 out of the 14 METTL family protein methyltransferases assessed. For each gene, the left panel shows z-scored protein abundance in matched normal and tumour tissue, and the right panel shows z-scored transcript abundance (TPM-derived) in tumour samples only (no matched normal transcriptomic samples were available in this dataset). Each point represents an individual sample.

To further investigate whether transcriptomic observations were reflected at the protein level in primary tumours, proteogenomic analyses were performed using data from the Clinical Proteomic Tumour Analysis Consortium (CPTAC) datasets (https://pdc.cancer.gov/), a public resource integrating matched proteomic and transcriptomic profiles from clinically annotated human cancers [60]. Colon adenocarcinoma (COAD) was selected as an initial case study owing to its prevalence and the growing interest in understanding environmental and lifestyle contributions to colon inflammation and progression towards cancer. Proteomic coverage proved to be the principal limiting factor, since of the fourteen METTL family members, only METTL11A and METTL13 were detected at the protein level in COAD, compared with nine detected transcriptomically (Figure 11). For these two proteomically detected proteins, a paired tumour-versus-normal comparison showed a modest reduction in tumour tissue for both METTL11A and METTL13, though neither reached statistical significance after correction (padj = 0.81 for both). Further analysis of expression by tumour stage, using all available transcriptomic and proteomic data, likewise revealed no statistically significant changes for any METTL across the four stages assessed (Figure S6). Although proteomic coverage was limited in COAD, these findings illustrate the practical challenges of evaluating low-abundance methyltransferases using current large-scale proteomic datasets and reinforce the importance of interpreting non-detection cautiously.

**Figure 12:**
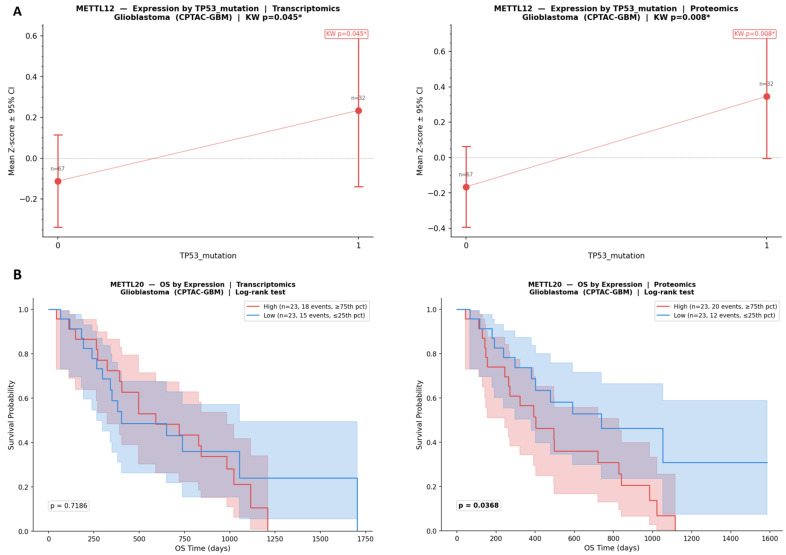
Summary of significant outputs from the CPTAC-GBM analyses for the METTL family protein methyltransferases. (**A**) Mean z-scored METTL12 expression stratified by TP53 mutation status (0 = wild-type, 1 = mutant) at the transcriptomic level (**left**) and proteomic level (**right**). METTL12 expression was significantly elevated in TP53-mutant tumours relative to wild-type at both layers. Points show mean z-score ± 95% CI. * *p* < 0.05. (**B**) Kaplan–Meier overall survival curves for patients stratified into high- (≥75th percentile) and low-expression (≤25th percentile) quartiles for METTL20, at the transcriptomic level (**left**) and proteomic level (**right**). High METTL20 expression, at protein but not at transcript expression, was associated with reduced overall survival, indicating that this prognostic association is detectable at the proteomic level only. Shaded bands represent 95% confidence intervals.

Given the limited findings in COAD, a contrasting tumour type was selected, one without a clear environmental aetiology and with a staging system grounded in a defined molecular biomarker rather than anatomical spread. Glioblastoma multiforme (GBM) best fulfilled these criteria. Coverage of the METTL family was markedly improved relative to COAD: 10 of 14 members were detected at the protein level and 13 of 14 at the transcript level (Figure S7). METTL9, METTL11B and METTL21A were detected transcriptomically only, while METTL23 remained undetected in both layers. The analysis examined METTL expression in relation to TP53 mutation status in GBM. METTL12 expression was significantly associated with TP53 mutation status at both the proteomic (*p* = 0.008) and transcriptomic (*p* = 0.045) levels, and it was the only family member to reach significance in both layers. METTL11A also showed a significant transcriptomic association (*p* = 0.017), though this was not mirrored at the protein level (Figure 12A and Figure S8). Next, Kaplan–Meier analysis of overall survival (OS) was performed by stratifying patients into high- and low-expression quartiles (≥75th and ≤25th percentile, respectively) for each METTL protein, using both transcriptomic and proteomic data. Of the family, METTL20 was the only protein to reach statistical significance and did so specifically at the protein level (log-rank *p* = 0.037, *n* = 23 per group): patients with high METTL20 protein expression showed reduced overall survival relative to those with low expression. The corresponding transcriptomic comparison for METTL20 was not significant (log-rank *p* = 0.719), indicating that this prognostic association is detectable at the proteomic level only (Figure 12B and Figure S9).

Overall, these integrative analyses demonstrate that protein-methylating METTL family members exhibit substantial heterogeneity across genomic, transcriptomic and proteomic layers, with no single family member emerging as a uniformly dysregulated, pan-cancer driver. Instead, distinct METTLs are prioritised as candidates of interest depending on the analytical layer examined: for example METTL13, METTL18, METTL21C, METTL21D and METTL23 as the most frequent genomically amplified; METTL9 as the most transcriptionally dynamic; METTL23, METTL21D and METTL11A as showing the most consistent sub-threshold dependency signal in CRISPR screens, contrasted with the apparent growth-promoting profile of METTL18 and METTL22; METTL12 as the protein most robustly linked to TP53 mutation status in GBM at both the transcript and protein level; and METTL20 as the only family member showing a significant association with overall survival, specifically at the protein level.

Critically, the poor and frequently inverse correlation between CCLE cell line and primary TCGA tumour expression data indicates that conclusions drawn from cell line models alone risk misrepresenting METTL biology in the in vivo primary disease, while the markedly incomplete proteomic coverage observed in COAD illustrates that absence of detection should not be interpreted as absence of biological relevance.

Taken together, these analyses using publicly available multi-omic datasets complement the currently limited functional literature by highlighting the divergence in expression among the protein-methylating METTL enzymes, in which biological significance is likely to be both context- and tissue-specific. Consistent with the observational nature of these analyses, these findings should be viewed as hypothesis-generating and require targeted, hypothesis-driven, functional experimental validation, particularly for METTL9, METTL12, METTL20 and METTL23, rather than continued reliance on correlative multi-omic screening alone to establish causal roles in tumour biology.

## 7. Current Knowledge Gaps

Despite recent progress in the characterisation of protein-methylating METTL enzymes, major questions remain unresolved. These gaps limit our understanding of their physiological and pathological roles, as well as their therapeutic potential. In particular, unresolved issues span: the incomplete mapping of protein substrates, the determinants and consequences of their distributive methylation activity, the lack of structural and functional data for a number of family members, and the lack of identified demethylases. Addressing these areas will be critical to move beyond descriptive findings and toward a deeper mechanistic and functional understanding of METTL biology. Bridging these gaps will require innovative experimental strategies and integrative approaches, which are outlined in the following section.

### 7.1. Unknown Protein Targets

For many METTL enzymes, only a handful of substrates have been identified, and in some cases, no orthogonally validated protein targets exist. Even among those with confirmed substrates (e.g., METTL21A–D, METTL13), the broader spectrum of possible interacting proteins remains poorly defined [44]. High-throughput proteomic screens and substrate-isolating approaches are required to uncover the full range of targets and pathways in which the protein-methylating METTLs are involved [61,62].

### 7.2. Unresolved Mechanistic Determinants

While distributive methylation by protein-methylating METTL enzymes has been observed, the precise factors determining whether a substrate is mono-, di-, or trimethylated remain unclear. Neither the structural nor the kinetic features that govern progression through these methylation states have been systematically elucidated, and the functional consequences of each methylation state are still largely unexplored. An equivalent investigation for the PRMT7 provided a unique perspective on which degree of methylation is added and potentially why [63]. Moreover, it is unknown whether protein-methylating METTL enzymes act constitutively or whether their activity is dynamically regulated in response to cellular signalling events. These unresolved determinants represent a central gap in understanding the biological logic of METTL-mediated methylation.

### 7.3. Missing Structural and Functional Data

Although all METTLs share the Rossmann-like fold and SAM-binding domain, crystal structures or cryo-EM data are missing or only partial for most protein-methylating members [35]. Without such information, predictions about substrate recognition, catalytic efficiency, or allosteric regulation remain speculative at best. The combination of structural and functional information has been critical in understanding other methyltransferase groups [2,64,65]. Moreover, functional redundancy or interplay among METTL enzymes has not been tested experimentally, leaving open the possibility that multiple enzymes may target overlapping protein sets under different conditions (despite this being unlikely given their uniqueness).

### 7.4. Unknown Demethylating Enzymes

Unlike histone methylation, where several demethylases have been characterised, no enzymes are currently known to reverse METTL-catalysed protein methylation [66,67,68]. While it is reasonable to presume that these modifications are reversed enzymatically, it is also possible that due to rapid target turnover, modulation of methyltransferase activity by itself is enough for regulation [62]. So far, neither the involvement of general demethylases such as LSD1 nor the existence of dedicated demethylases has been systematically investigated. Understanding how METTL-mediated methylation is reversed is critical for understanding the dynamic regulation of METTL activity and for evaluating their potential as therapeutic targets.

## 8. Future Directions

Despite significant progress over the past decade, research into protein-methylating METTL enzymes remains at a relatively early stage compared with other methyltransferase families. The identification of substrates, elucidation of catalytic mechanisms, and understanding of biological consequences are still fragmented, limiting our ability to define their contribution to physiological and pathological roles. Moving forward, a combination of proteomics, cell biology, structural biology, and genetic manipulation tools and techniques are poised to close these gaps. Importantly, linking the enzymes’ biochemical activity to disease phenotypes will be critical, not only for mechanistic insight but also for the potential exploitation of METTLs as biomarkers and therapeutic targets. The following sub-sections highlight targeted experimental and analytical approaches for studying protein-methylating METTLs, as well as the therapeutic and diagnostic opportunities that could arise from a deeper understanding of their biology.

### 8.1. Methodological Advances to Study Protein-Methylating METTLs

A deeper mechanistic understanding of protein-methylating METTL enzymes requires cutting-edge targeted experimental and analytical approaches. Several lines of investigation are particularly promising, spanning proteomics, spatial biology, structural studies, genetic tools, and functional assays. The output from these methodologies taken together will help to address unresolved knowledge gaps identified in Section 7, including substrate identification, structural specificity, and dynamic regulation.

#### 8.1.1. Proteomic Mapping of Methylation Events

Comprehensively identifying substrates of protein-methylating METTLs remains one of the most pressing challenges. Advances in methyl-specific enrichment strategies, such as immunoaffinity purification of methylated peptides or chemical derivatisation approaches, when coupled with high-resolution mass spectrometry, now allow global detection of lysine methylation events at scale [69,70]. These workflows provide an unbiased means of discovering novel substrates and quantifying methylation stoichiometry across conditions. Importantly, previous studies have shown that most identified METTL targets are non-histone proteins, often with structural or enzymatic functions, highlighting the utility of proteomic surveys to uncover unexpected biology. Integration with quantitative proteomics, including SILAC or TMT labelling, enables comparative analysis of substrate methylation upon METTL perturbation, providing functional context [17,71]. However, distinguishing direct METTL substrates from secondary downstream effects remains a limitation, emphasising the need to combine proteomic mapping with orthogonal validation strategies such as structural studies or targeted mutagenesis.

#### 8.1.2. Spatial Proteomics

The functional role of a METTL enzyme is intimately linked to its sub-cellular localisation, which determines substrate accessibility. Emerging spatial proteomics strategies enable high-resolution mapping of protein localisation and interaction networks. The employment of approaches such as sub-cellular fractionation combined with quantitative MS, or proximity-labelling approaches such as bioID and APEX2 make it possible to chart METTL activity in situ [72,73,74]. Such methods will clarify whether substrate selectivity arises from intrinsic recognition motifs, or from spatial compartmentalisation within the cell [75,76,77,78].

#### 8.1.3. Structural Biology

Resolving the three-dimensional structures of protein-methylating METTL enzymes in complex with their substrates is critical to understanding the molecular determinants of target recognition and catalytic specificity [79]. Techniques such as cryo-electron microscopy and X-ray crystallography can capture substrate-bound complexes, revealing how conserved motifs, active site residues, and substrate-binding pockets contribute to methylation [35]. Integrating structural insights with molecular dynamics simulations may further uncover how differential substrate specificity is achieved among closely related METTL family members [80,81]. This would provide critical information towards the design of selective inhibitors or stabilisers. In fact, docking experiments have been used to develop the METTL9 inhibitor METTL9i that binds within the SAM-binding pocket [82], the METTL10 inhibitor LZQ-02-023-01 [31] and the METTL11A inhibitor GD433 that is a competitive inhibitor for the substrate-binding site [83].

#### 8.1.4. Genetic Tools

Functional investigations of protein-methylating METTLs require targeted manipulation in biologically relevant systems. CRISPR/Cas9-mediated knockout and knock-in approaches provide powerful strategies to define physiological roles, allowing the evaluation of the outcomes following complete METTL enzyme loss or enzymatic inactivation, when catalytically inactive mutants are generated [35,84,85,86,87]. Nevertheless, RNAi-mediated knockdown might be a more useful alternative in contexts where complete knockout is not feasible [80]. For the purpose of increasing enzyme expression, both transient and stable inducible overexpression strategies (such as the tetracycline-controlled expression system) are viable options, with the intention of attempting to increase overall enzyme activity when applied in either cultured cells or animal models. These complementary tools allow the investigation of whether altering intracellular METTL levels result in changes in substrate methylation, protein stability, or cellular signalling pathways [88]. When combined with proteomic quantification and structural information, genetic manipulation allows the collection of direct evidence to link METTL activity to disease phenotypes. Such evidence would complement biochemical data for connecting molecular mechanisms with pathological outcomes.

#### 8.1.5. Activity Assays

Sensitive assays are essential to monitor the activity of protein-methylating METTLs in both biochemical (in vitro) and cellular (in vivo) contexts [89]. Currently it is quite difficult to determine methyltransferase activity in vitro and almost impossible to do so in vivo. The inability to follow and quantify accurately dynamic methylation events highlights the need for the development of robust in vitro assays, based on radio-labelled methyl donor incorporation or fluorescence-based reporters [90,91]. In parallel, developing in vivo reporters, for collecting real time methylation status in live cell cultures, would enable the study of METTL activity under physiological conditions [92,93]. Developing quantitative activity assays will not only advance mechanistic studies but also serve as essential tools in the screening and optimisation of small-molecule modulators in drug discovery workflows [94].

Coupled with proteomic quantification, structural information, and genetic modulation, activity assays provide an experimental toolkit for connecting enzyme activity to downstream cellular outcomes and therapeutic potential [12].

### 8.2. Therapeutic and Diagnostic Implications

The growing recognition of protein-methylating METTLs as regulators of cellular physiology raises the possibility that they could serve as both clinical biomarkers and therapeutic targets [95,96]. Their high substrate specificity, emerging links to cancer and other disorders, and potential integration within broader epiproteomic networks make them attractive candidates for translational exploration.

#### 8.2.1. Target Validation

A critical step in translating the biochemical knowledge about protein-methylating METTLs into a therapeutic context is the validation of the individual enzymes as clear contributors to a specific disease phenotype. While associations between certain protein-methylating METTLs and cancer progression or other disorders are increasingly reported, establishing direct mechanistic links remains limited [96]. Functional studies employing loss-of-function (CRISPR/Cas9, RNAi) or gain-of-function (transient or stable tetracycline inducible overexpression) approaches, in both in vitro and in vivo models, are necessary to determine whether perturbation of specific protein-methylating METTLs alters disease-relevant pathways [87]. Understanding which protein-methylating METTL is linked to a particular pathological phenotype will drive interest in developing small-molecule inhibitors and targeted therapeutic strategies. Moreover, insights into how protein-methylating METTL perturbations alter cellular signalling, protein stability, or substrate methylation in specific tissues or cell types may reveal context-dependent cellular weaknesses, supporting precision medicine approaches. Integrating biochemical, cellular, and patient data will be key to distinguishing protein-methylating METTLs that functionally impact a particular cancer or condition from those with minor roles [95].

#### 8.2.2. Biomarker Potential

The expression patterns and substrate-specific methylation profiles of protein-methylating METTLs provide opportunities for biomarker development. Altered METTL expression or abnormal methylation states may serve as prognostic indicators, particularly in cancer, where correlations with tumour stage, metastatic potential, or therapeutic response and chemoresistance have been suggested [96]. In addition to protein levels, measuring methylation marks on specific substrates could offer functional readouts of enzymatic activity in patient samples. Coupling such quantitative proteomic assessment with transcriptomic profiling may enable robust stratification of patient populations and inform individualised treatment strategies. However, the lack of standardisation across detection methods and the current inability to validate in large cohorts limits the clinical utility of METTLs as biomarkers.

#### 8.2.3. Drug Discovery

Once individual protein-methylating METTLs are validated as disease-relevant and measurable in patient contexts, the next step is the pursuit of therapeutic modulators. Structure-guided design and high-throughput virtual screening approaches can be leveraged to discover inhibitors (to suppress aberrant methylation that drives oncogenic processes) or stabilisers (to prolong the activity of METTLs with protective roles) of these enzymes [97]. Computational modelling, including molecular docking and dynamic simulations, can accelerate the identification of potential compounds and predict binding affinities and selectivity, particularly given the diverse substrate-binding pockets of METTL family members. Combining computational predictions with biochemical and cellular assays allows iterative refinement of candidate molecules, increasing the likelihood of identifying potent and selective modulators. Such integrated strategies are essential for translating mechanistic insights into therapeutically actionable interventions, with the ultimate goal of modulating METTL activity in disease-relevant contexts. To achieve this, careful evaluation of off-target effects and pharmacokinetic properties will be required to ensure that METTL-targeting compounds can transition from experimental tools to true clinical candidates [7].

#### 8.2.4. Epigenetic Context

In light of these insights, therapeutic and diagnostic perspectives must be framed within the broader regulatory framework of cellular signalling [12,98]. Protein-methylating METTLs put down methylation marks that engage in dynamic crosstalk with other post-translational modifications, such as acetylation, phosphorylation, and ubiquitination, creating a complex regulatory network that shapes protein stability, localisation, and function [99,100]. Mapping how METTL-dependent methylation interacts with these modifications could uncover synergistic or competitive relationships of therapeutic significance [12]. For example, methylation by protein-methylating METTLs may block ubiquitination or modulate the possibility of phosphorylation, influencing turnover or signalling output, influencing the effectiveness of standard cancer therapies. Mapping these epiproteomic crosstalk networks in disease-relevant contexts will not only clarify METTL function but may also reveal opportunities for multi-target intervention, where using combination therapies including molecules that modulate METTL activity together with other epigenetic regulators could enhance therapeutic benefit as has been obtained for other methyltransferases [7].

## 9. Conclusions

Over the past decade, considerable progress has been made in defining the functions of protein-methylating METTL enzymes. These enzymes stand out among methyltransferases for their unusually high substrate specificity, often acting on a single protein or a closely related group of substrates. The resulting methylations have diverse consequences for protein stability, localisation, and activity, highlighting their role as precise regulators of cellular processes rather than broad epigenetic switches.

Despite this progress, our understanding of the METTL family remains incomplete. The biological significance of many identified methylation marks is still poorly defined, how their 3D structures dictate substrate recognition remains unresolved for most members, and no demethylases have yet been linked to reversing these modifications. Furthermore, the evolutionary origins and justification for such finely tuned substrate specificity, and the full spectrum of diseases in which they are dysregulated, remain open questions.

Future research must therefore address four central challenges: (1) clarifying the functional roles of METTL-mediated methylations in human biology, (2) identifying and validating new substrates, (3) uncovering the mechanisms and reversibility of these modifications, and (4) delineating the disease contexts in which they play critical roles. Meeting these challenges will require the integration of advanced proteomics, structural and computational biology, as well as functional investigations spanning biochemical in vitro assays, cell culture models, animal model organisms and human clinical samples.

As functional and multi-omic evidence linking protein-methylating METTLs to cancer and other disorders continues to grow, it is becoming increasingly clear that they are not negligible actors but may represent important modulators of cellular physiology. Filling the current knowledge gaps will not only advance our understanding of methylation biology but also open new opportunities for therapeutic and diagnostic applications.

## Figures and Tables

**Table 1:** METTL family members in humans and their reported target biomolecules (Data retrieved from https://www.uniprot.org/ [18]).

Name	ID	Synonyms	Target
METTL 1	Q9UBP6	C12orf1, TRM8, TRMT8, YDL201w	tRNA
METTL 2A	Q96IZ6	FLJ12760, METTL2	tRNA
METTL 2B	Q6P1Q9	FLJ11350, METL, METTL2, PSENIP1, METTL2A	tRNA
METTL 3	Q86U44	M6A, MT-A70, Spo8, HMETTL3, MTA70, IME4	mRNA
METTL 4	Q8N3J2	FLJ23017, HsT661	mRNAs, DNA
METTL 5	Q9NRN9	HSPC133, MRT72	rRNA
METTL 6	Q8TCB7	MGC24132	tRNA
METTL 7A	Q9H8H3	DKFZP586A0522, AAM-B, AAMB	alkyl thiols
METTL 7B	Q6UX53	ALDI, MGC17301	alkyl thiols
METTL 8	Q9H825	FLJ13984, TIP	mRNA
METTL 9	Q9H1A3	DREV1, DREV, CTB-31N19.3, CGI-81, PAP1	Protein
METTL 10	Q5JPI9	C10orf138, EEF1AKMT2, Efm4	Protein
METTL 11A	Q9BV86	AD-003, C9orf32, HOMT1A, NTM1A, NRMT1, NRMT	Protein
METTL 11B	Q5VVY1	C1orf184, HOMT1B, NTM1B, NRMT2	Protein
METTL 12	A8MUP2	U99HG, CS-KMT	Protein
METTL 13	Q8N6R0	CGI-01, KIAA0859, DFNB26, DFNB26M, EEF1AKNMT, FEAT, 5630401D24Rik	Protein
METTL 14	Q9HCE5	KIAA1627, HMETTL14	mRNA
METTL 15	A6NJ78	FLJ33979, METT5D1	rRNA
METTL 16	Q86W50	METT10D, MGC3329	snRNA and mRNA
METTL 17	Q9H7H0	FLJ20859, METT11D1	rRNA
METTL 18	O95568	AsTP2, C1orf156, HPM1, MGC9084, ASTP2	Protein
METTL 19	Q8IYL2	C4orf23, FLJ35725, TRM44, TRMT44	tRNA
METTL 20	Q8IXQ9	C12orf72, DKFZp451L235, MGC50559, ETFB-KMT	Protein
METTL 21A	Q8WXB1	FAM119A, HCA557b, HSPA-KMT, LOC151194, HCA557B	Protein
METTL 21B	Q96AZ1	DKFZP586D0919, FAM119B, EEF1AKMT3, HCA557A	Protein
METTL 21C	Q5VZV1	C13orf39, LOC196541	Protein
METTL 21D	Q9H867	C14orf138, VCP-KMT	Protein
METTL 21E	N.A.	N.A.	N.A. (Pseudogene in humans)
METTL 22	Q9BUU2	C16orf68, FLJ12433, MGC2654, LP8272	Protein
METTL 23	Q86XA0	C17orf95, LOC124512, MRT44	Protein
METTL 24	Q5JXM2	C6orf186, dJ71D21.2	Unknown
METTL 25A	Q8N6Q8	C12orf26, FLJ22789	Unknown
METTL 25B	Q96FB5	C1orf66, RRNAD1	Unknown
METTL 26	Q96S19	JFP2, C16orf13, MGC13114	Unknown
METTL 27	Q8N6F8	WBSCR27	Unknown

**Table 2:** METTL family protein methyltransferase targets.

Name	Amino Acid	Protein Targets	Reference
METTL 9	Histidine	Proteins with a His-x-His (HxH) motif, e.g., NODO1, NCLN, SLC39A5	[28,29]
METTL 10	Lysine	EEF1A, PIAS3	[30,31]
METTL 11A	Lysine	N-terminus of proteins with N-terminal motif [Ala/Gly/Pro/Ser]-Pro-Lys with cleaved initiator Met, e.g., RCC1, DDB2, CENPA, RPL12, PRMT6	[32]
METTL 11B	Lysine	N-terminus of proteins with N-terminal motif [Ala/Gly/Pro/Ser]-Pro-Lys with cleaved initiator Met, e.g., RCC1, DDB2, CENPA, RPL12, PRMT6	[33]
METTL 12	Lysine	Citrate Synthase	[34]
METTL 13	Lysine	Elongation factor 1-alpha (EEF1A1 and EEF1A2)	[35]
METTL 18	Histidine	RPL3	[36]
METTL 20	Lysine	Electron Transfer Flavoprotein Beta Subunit	[37]
METTL 21A	Lysine	HSPA1/2/5/6/8	[38]
METTL 21B	Lysine	EEF1A1, EEF1A2	[35]
METTL 21C	Lysine	AARS1, VCP/p97	[39]
METTL 21D	Lysine	VCP/p97	[26]
METTL 22	Lysine	Kin17	[19]
METTL 23	Arginine	Histone H3	[40]

**Table 3:** Sub-cellular localisation of METTL family protein methyltransferases.

Name	Sub-Cellular Localisation	Reference
METTL 9	Mitochondria and Endoplasmic reticulum	[28]
METTL 10	Nucleus and Cytoplasm	[30]
METTL 11A	Nucleus	[32]
METTL 11B	Nucleus	[32]
METTL 12	Mitochondria	[41]
METTL 13	Cytoplasm, Mitochondria and Nucleus	[42]
METTL 18	Nucleus	[36]
METTL 20	Mitochondria; Cytoplasm	[19,37]
METTL 21A	Cytoplasm	[19]
METTL 21B	Cytoplasm and Centrosome	[43]
METTL 21C	Nucleus	[19]
METTL 21D	Cytoplasm	[19]
METTL 21E	Unknown	N.A.
METTL 22	Nucleus	[19]
METTL 23	Cytoplasm and Membrane	[19]

**Table 4:** Degree of methylation added by METTL family protein methyltransferases.

Name	Degree of Methylation	Reference
METTL 9	Monomethylation (1-methylhistidine)	[28]
METTL 10	Trimethylation	[30]
METTL 11A	Mono-, Di-, Trimethylation of Ala, Gly or Ser. Mono-, Dimethylation of Pro.	[33]
METTL 11B	Monomethylation	[33]
METTL 12	Trimethylation	[34]
METTL 13	Trimethylation	[35]
METTL 18	Monomethylation (3-methylhistidine)	[36]
METTL 20	Dimethylation, Trimethylation	[37]
METTL 21A	Trimethylation	[38]
METTL 21B	Mono-, Di-, Trimethylation	[43]
METTL 21C	Monomethylation	[26]
METTL 21D	Trimethylation	[26]
METTL 22	Trimethylation	[19]
METTL 23	Di for Arg (probably asymmetrical)	[40]

**Table 5:** Cell types presenting the highest RNA and protein expression for the METTL family protein methyltransferases (data retrieved from The Human Protein Atlas [45]).

Name	RNA Expression Level	Protein Expression Level
METTL 9	Renal tubules	Various
METTL 10	Renal tubules	Various
METTL 11A	Spermatids	Various
METTL 11B	N.A.	N.A.
METTL 12	Alveoles and bronchioles	Renal tubules, Seminiferous tubules, and trophoblasts
METTL 13	Spermatids	Various
METTL 18	Renal tubules	Parathyroid gland
METTL 20	Renal tubules	Various
METTL 21A	Small intestine crypts	Various
METTL 21B	Monocytes	N.A.
METTL 21C	Spermatids	N.A.
METTL 21D	Keratinocytes	Various
METTL 22	Spermatids	Parathyroid gland, adrenal gland, salivary gland, and epididymis
METTL 23	Spermatids	N.A.

## Data Availability

No new data were created or analyzed in this study. Data sharing is not applicable to this article.

## Supplementary Materials

The following supporting information can be downloaded at: https://www.mdpi.com/article/10.3390/ijms27146532/s1.

## Abbreviations

The following abbreviations are used in this manuscript:

aa	Amino acid
aRme2	Asymmetric dimethylarginine
ATP	Adenosine triphosphate
CCLE	Cancer Cell Line Encyclopaedia
CERES	Computational correction of copy-number effect for CRISPR-Cas9 essentiality screens
CI	Confidence interval
COAD	Colon adenocarcinoma
CPTAC	Clinical Proteomic Tumour Analysis Consortium
CRC	Colorectal cancer
CRISPR	Clustered Regularly Interspaced Short Palindromic Repeats
DNA	Deoxyribonucleic acid
GBM	Glioblastoma multiforme
GISTIC	Genomic Identification of Significant Targets in Cancer
Hme	Methylhistidine
HR	Hazard ratio
ID	Intellectual disability
Kme1	Monomethyllysine
Kme2	Dimethyllysine
Kme3	Trimethyllysine
KMT	Lysine methyltransferase
LC-MS/MS	Liquid chromatography–tandem mass spectrometry
log_2_	Base-2 logarithm
MC3	Multi-Centre Mutation Calling in Multiple Cancers
METTL	Methyltransferase-like
mRNA	Messenger RNA
Nπ	Pros nitrogen of the histidine imidazole ring
Nτ	Tele nitrogen of the histidine imidazole ring
OS	Overall survival
padj	Adjusted *p*-value
PDB	Protein Data Bank
PKMT	Protein lysine methyltransferase
PMT	Protein methyltransferase
PRMT	Protein arginine methyltransferase
PTM	Post-translational modification
Rme1	Monomethylarginine
RNA	Ribonucleic acid
rRNA	Ribosomal RNA
RSEM	RNA-Seq by Expectation-Maximisation
SAH	S-Adenosylhomocysteine
SAM	S-Adenosyl-L-methionine (S-Adenosylmethionine)
SD	Standard deviation
SET	Su(var)3-9, Enhancer-of-zeste and Trithorax
sRme2	Symmetric dimethylarginine
TCGA	The Cancer Genome Atlas
TMT	Tandem Mass Tag
TP53	Tumour protein p53
TPM	Transcripts per million
UCEC	Uterine corpus endometrial carcinoma
VCP	Valosin-containing protein
WGS	Whole-genome sequencing
z-score	Standardised score
