# Supplementary material for: Decoding Protein-Methylating METTLs in Humans: Structural, Functional, and Disease Insights over the Past Decade
Source: Int J Mol Sci. 2026 Jul 22;27(14):6532. doi: 10.3390/ijms27146532 (PMC13410288; doi:10.3390/ijms27146532)
Supplement: Supplementary file 1 [file ijms-27-06532-s001.zip › ijms-4423138 supplementary figures.pdf]

Supplementary Figures

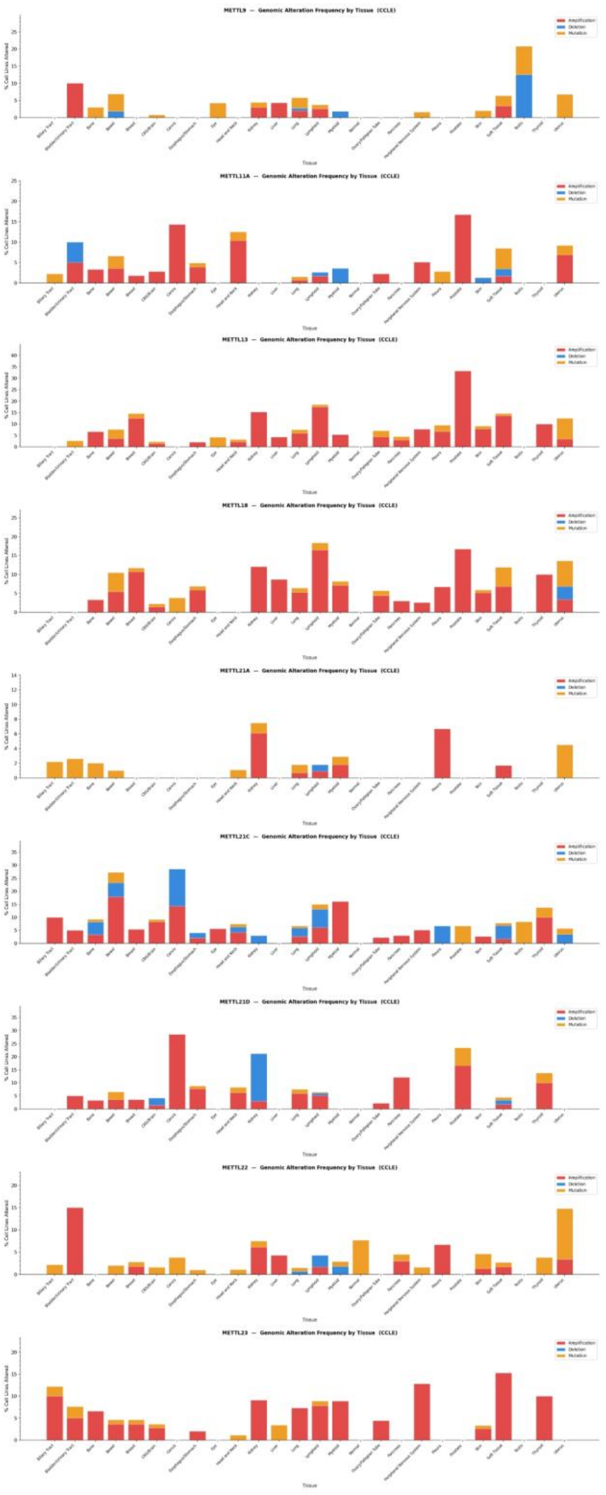

Supplementary Figure S1. Genomic alteration frequency for the METTL family protein methyltransferases across CCLE cell lines, grouped by tissue. For each gene, stacked bar plots show the percentage of cell lines per tissue harbouring a genomic alteration, presented as somatic mutations (orange), amplifications (red) and deletions (blue).

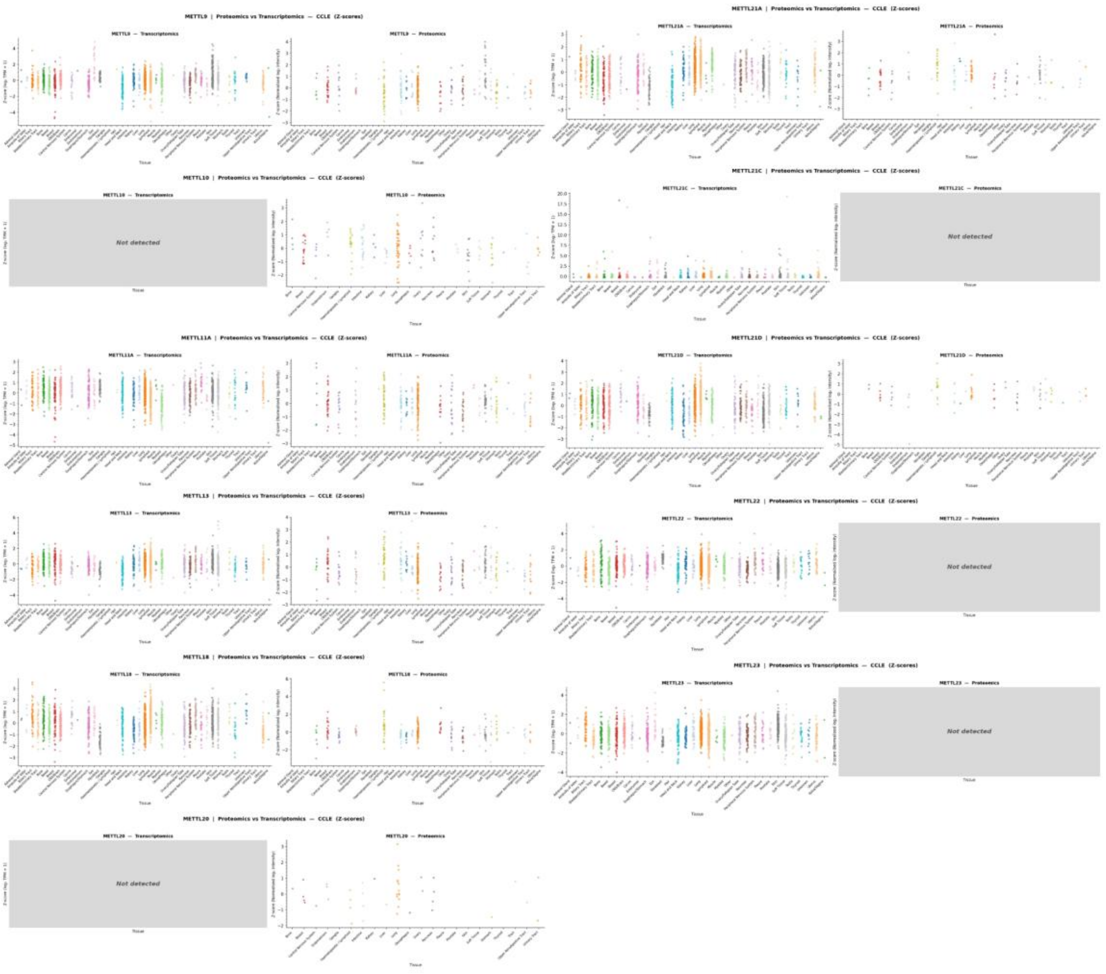

Supplementary Figure S2. Side-by-side transcriptomic and proteomic expression profiles for the METTL family protein methyltransferases across CCLE cell lines, grouped by tissue. A) Transcriptomic expression strip-plots where each point represents the z-scored log_2(TPM+1) RNA expression value for an individual cell line, relative to the pan-tissue mean for that gene, coloured by tissue. B) Proteomic expression strip-plots where each point represents the z-scored, normalised log_2 TMT-based protein intensity for an individual cell line, coloured by tissue. Greyed-out plots are depicted where data was not available in CCLE.

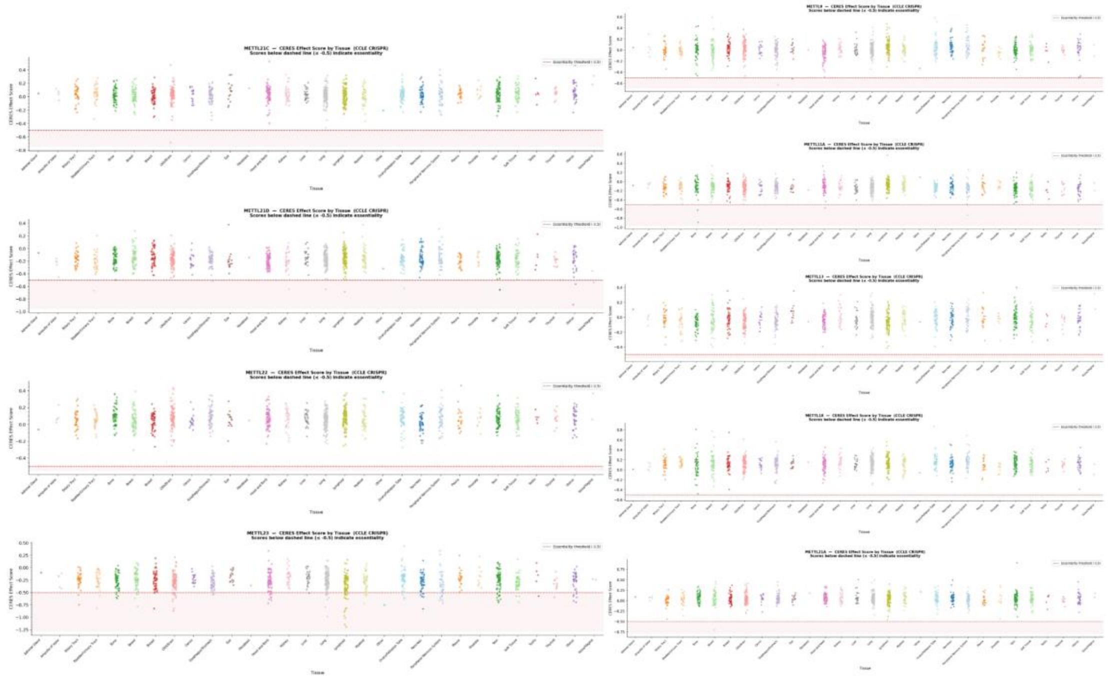

Supplementary Figure S3. CERES dependency effect score profiles for the METTL family protein methyltransferases across CCLE cell lines, grouped by tissue. Each point represents the CERES effect score for an individual cell line, derived from DepMap CRISPR knockout screens. The dashed red line marks the essentiality threshold (CERES ≤ −0.5); shaded pink regions indicate the essentiality zone, where knockout produces a growth-suppressive (essential) phenotype. Scores near zero indicate no fitness effect upon knockout; positive scores indicate a growth advantage. METTL proteins not screened in this dataset are not shown.

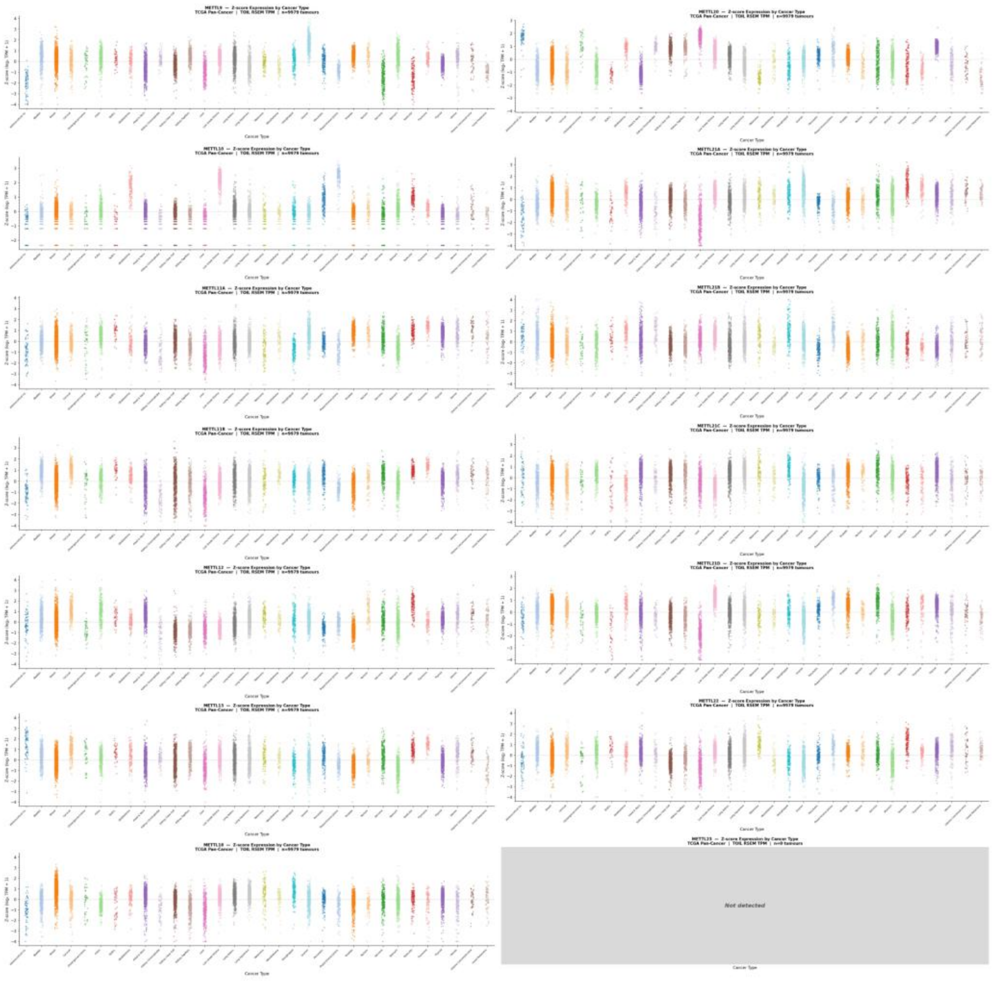

Supplementary Figure S4. Transcriptomic expression profiles for the METTL family protein methyltransferases across 33 TCGA pan-cancer tumour types (TOIL RSEM, n = 9,979 tumours). Transcriptomic expression strip-plots where each point represents the z-scored $\log_2$(TPM+1) RNA expression value for an individual tumour sample, relative to the pan-cancer mean for that gene, coloured by cancer type. METTL23 was not detected in this dataset (n = 0) and is shown as a blank panel.

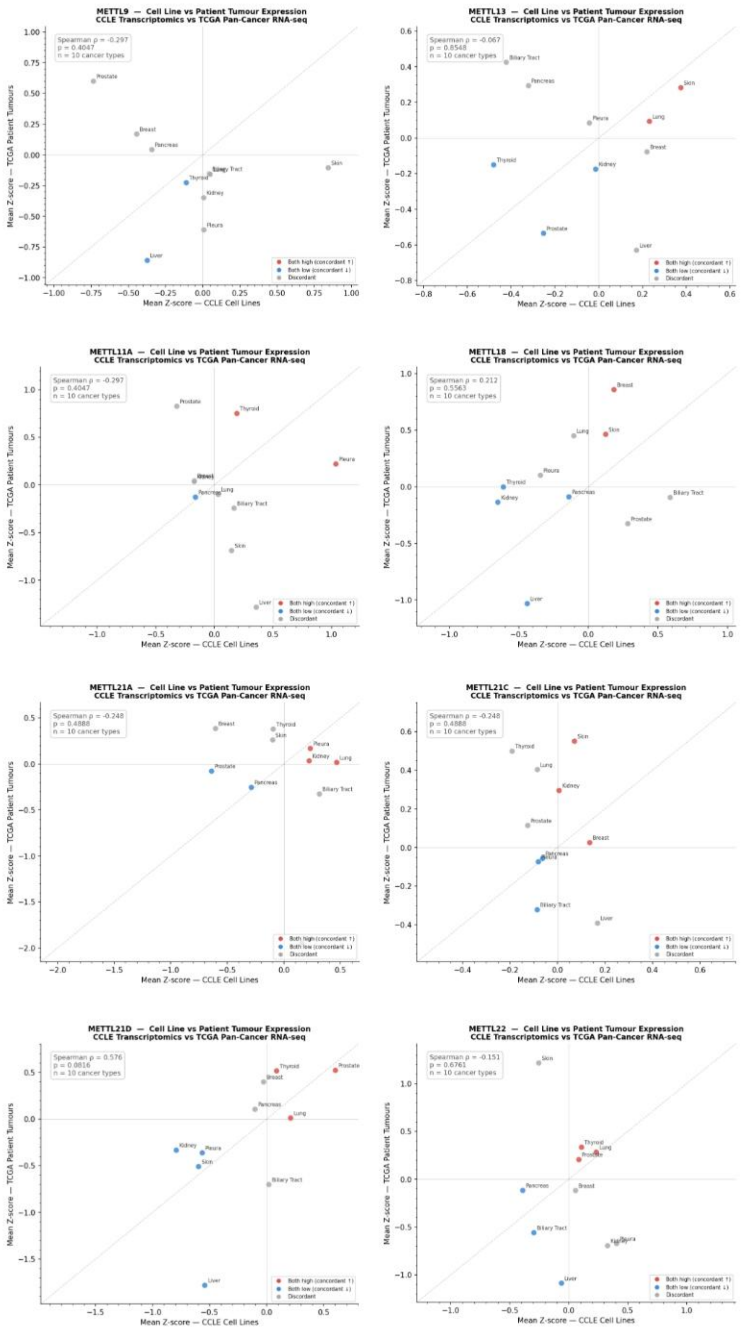

Supplementary Figure S5. Concordance between CCLE cell line and TCGA patient tumour transcriptomic expression for the subset of METTL family protein methyltransferases detected in both datasets. For each gene, points represent matched cancer types/tissues (n = 10), plotted by mean expression z-score in CCLE cell lines (x-axis) against mean expression z-score in TCGA patient tumours (y-axis); the diagonal line indicates perfect concordance. Points are coloured by concordance category: red indicates cancer types with elevated expression in both datasets ("both high"), blue indicates reduced expression in both datasets ("both low"), and grey indicates discordant direction between cell lines and tumours. Spearman correlation coefficients (ϱ) and associated p-values for each gene are reported in the inset text.

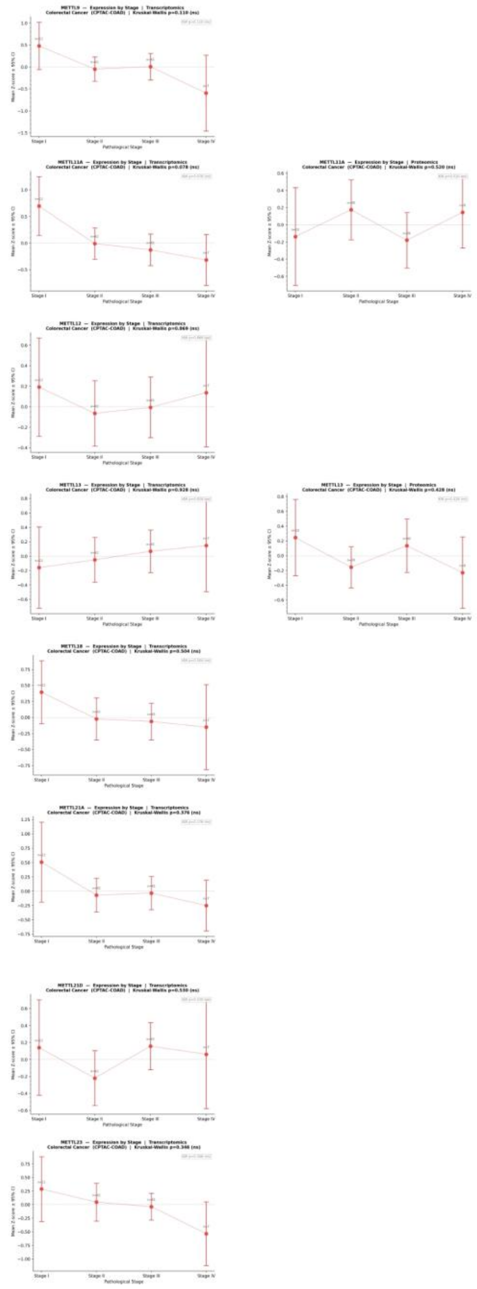

Supplementary Figure S6. Expression of METTL family protein methyltransferases across pathological tumour stage (Stage I–IV) in colon adenocarcinoma (CPTAC-COAD). Transcriptomic stage-stratified expression is shown for 8 of the 14 METTL family protein methyltransferases detected in this dataset, while proteomic stage-stratified expression is shown only for 2 of the 14 proteins. For each gene/modality, points represent the mean z-scored expression value per stage, with error bars showing 95% confidence intervals and sample size (n) per stage labelled above each point. Group differences across stages were assessed by Kruskal-Wallis test; no comparison reached statistical significance (all p > 0.05, ns), indicating no detectable association between METTL expression and pathological stage at either the transcript or protein level in this cohort.

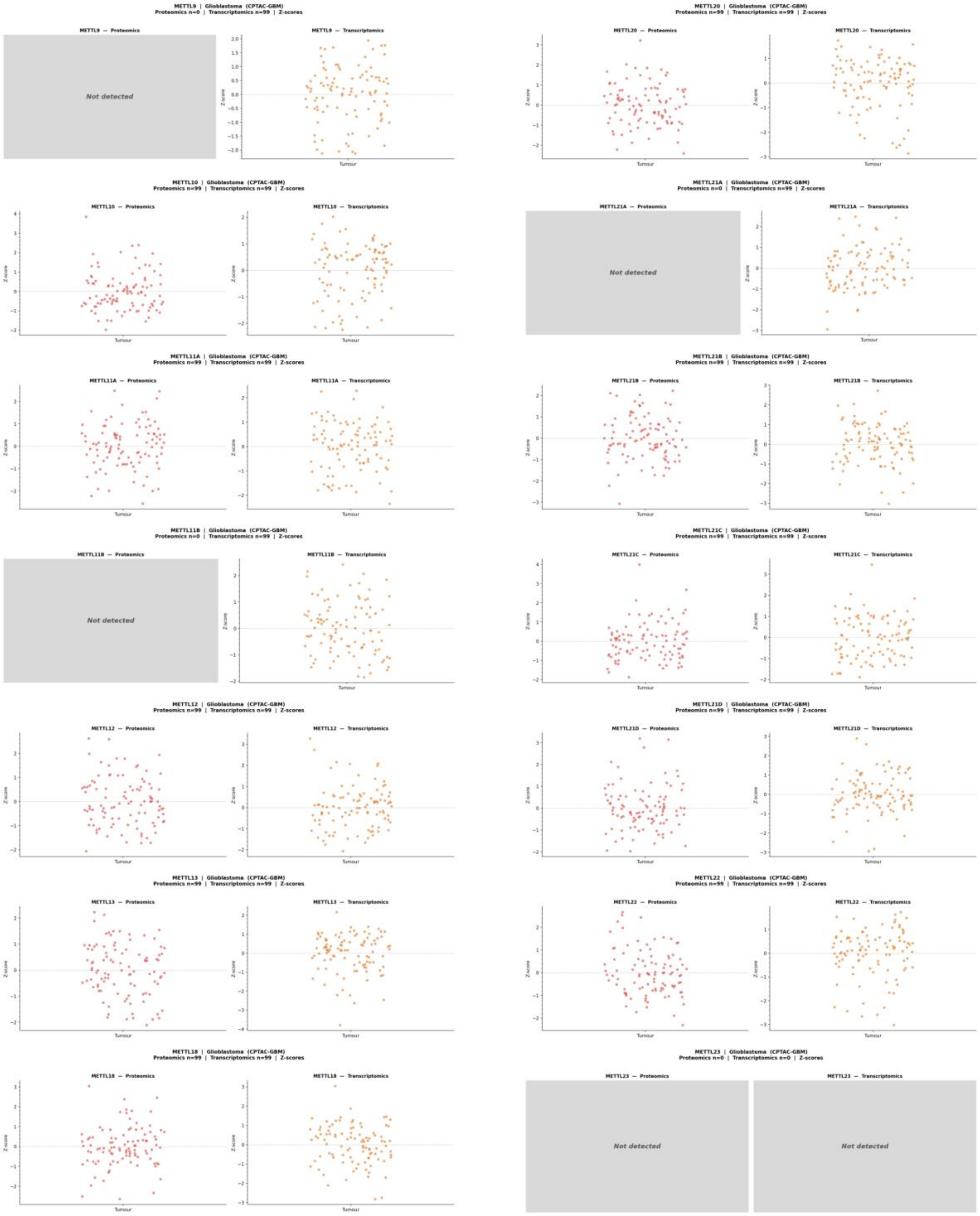

Supplementary Figure S7. Proteomic and transcriptomic expression of the METTL family protein methyltransferases (all 14 members) in glioblastoma patient tumours, from the CPTAC dataset (CPTAC-GBM). For each gene, the left panel shows z-scored protein abundance and the right panel shows z-scored transcript (TPM-derived) abundance, for tumour samples only (no matched normal samples were available in this dataset). Each point represents an individual sample. Grey panels indicate proteins that were not detected.

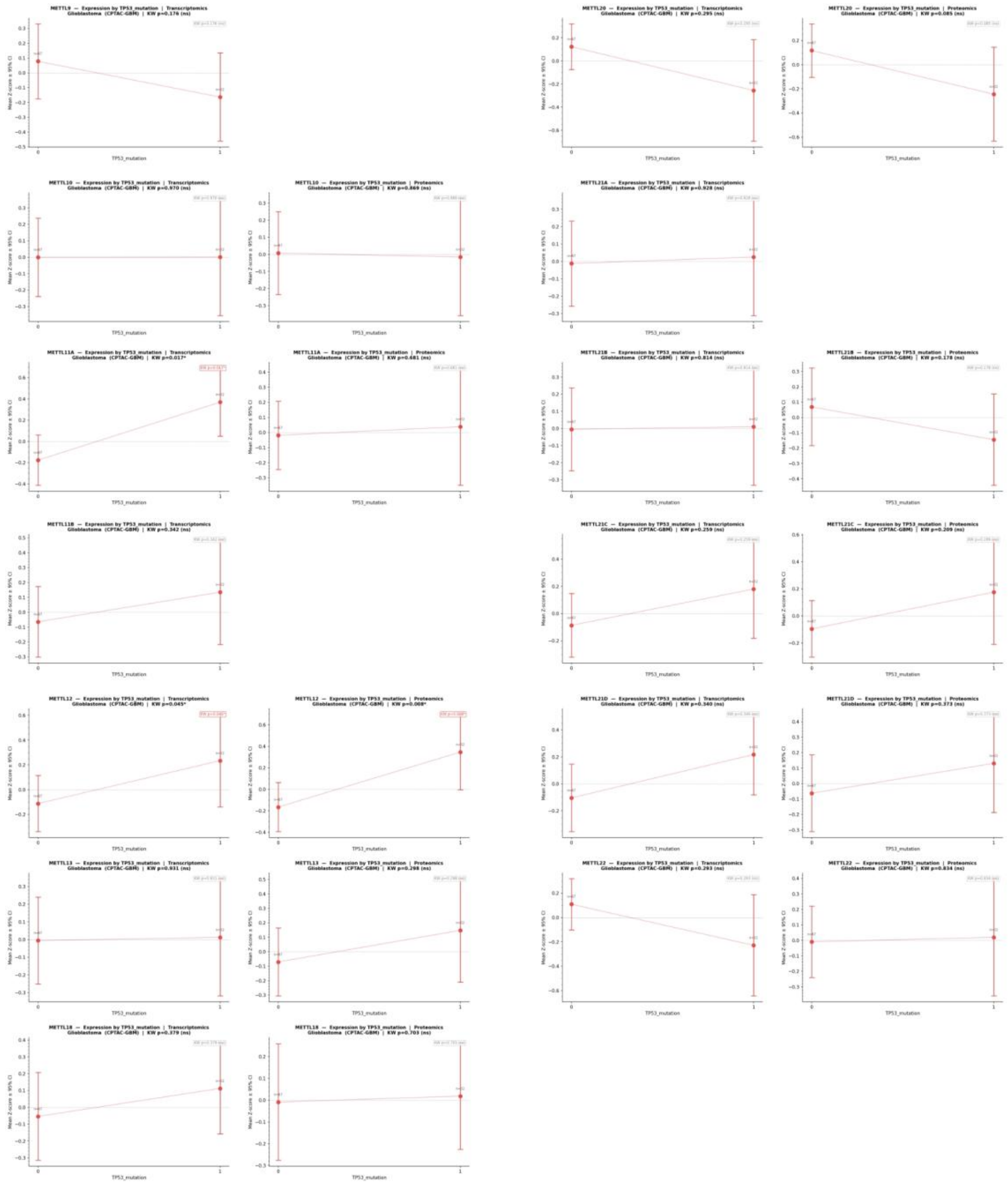

Supplementary Figure S8. Expression of METTL family protein methyltransferases by TP53 mutation status in glioblastoma (CPTAC-GBM). Transcriptomic comparisons are shown for 13 of 14 METTL family protein methyltransferases detected in this dataset. Proteomic comparisons are shown for 10 of 14 members (METTL23 was not detected at either omics layer). For each gene/modality, points represent the mean z-scored expression value per TP53 status group, with error bars showing 95% confidence intervals and sample size (n) labelled above each point. Group differences were assessed by Kruskal-Wallis test. METTL11A (transcriptomic, p=0.017) and METTL12 (transcriptomic, p=0.045; proteomic, p=0.008) were the only comparisons to reach statistical significance; METTL12 was the sole family member significant at both omics layers. All remaining comparisons were non-significant (p>0.05, ns).

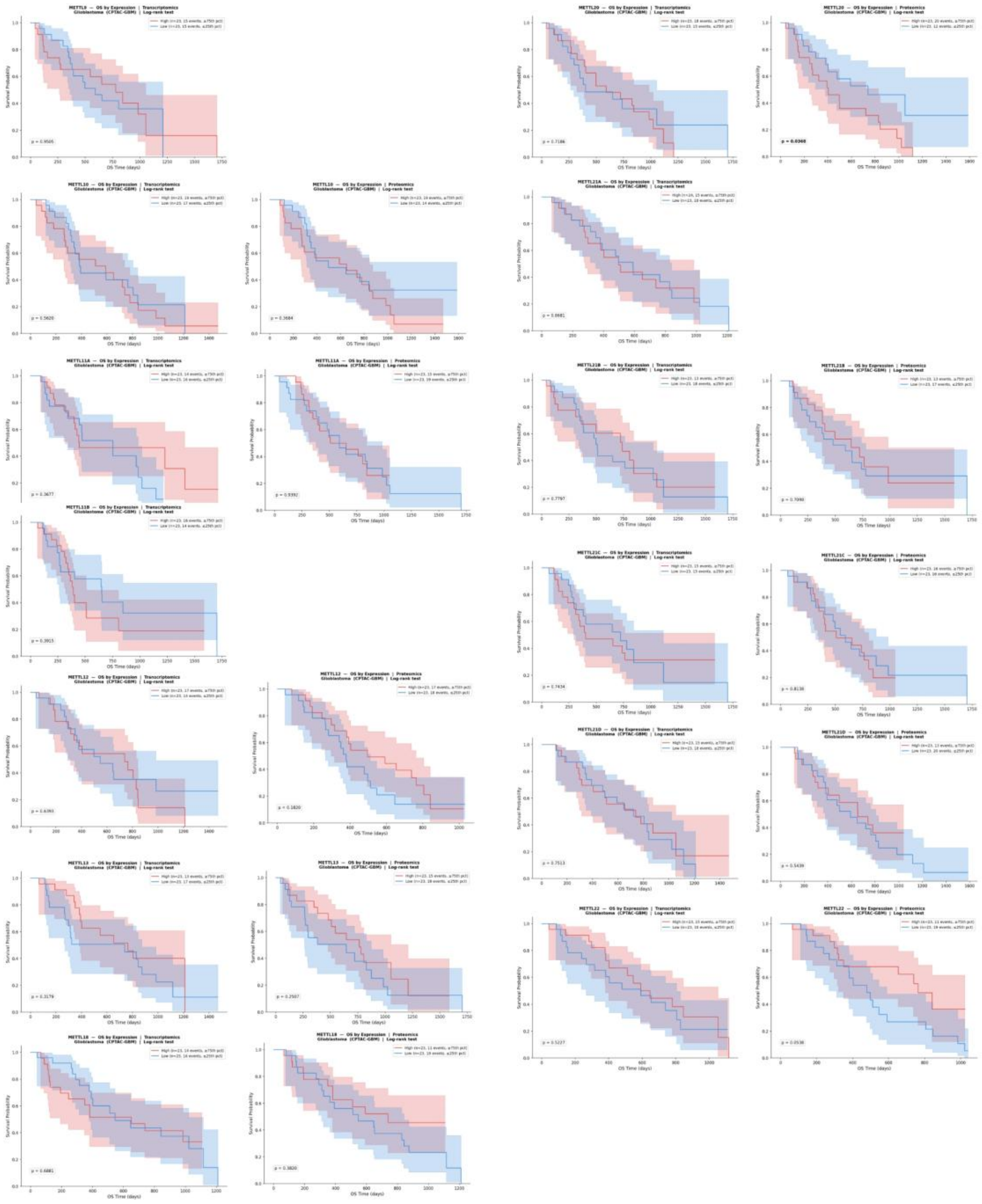

Supplementary Figure S9. Kaplan-Meier overall survival curves for glioblastoma patients (CPTAC-GBM), stratified by high- (≥75th percentile) versus low-expression (≤25th percentile) groups for each METTL family protein methyltransferase. Transcriptomic comparisons are shown for 13 of 14 METTL family members detected in this dataset. Proteomic comparisons are shown for 10 of 14 members (METTL23 was not detected at either omics layer). Shaded bands represent 95% confidence intervals; log-rank test p-values are annotated on each plot. METTL20 protein expression was the only comparison to reach statistical significance ($p=0.0368$), with high expression associated with reduced overall survival. All other comparisons were non-significant ($p>0.05$).
