# Supplementary material for: Decoding Protein-Methylating METTLs in Humans: Structural, Functional, and Disease Insights over the Past Decade
Source: Int J Mol Sci. 2026 Jul 22;27(14):6532. doi: 10.3390/ijms27146532 (PMC13410288; doi:10.3390/ijms27146532)
Supplement: Supplementary file 1 [file ijms-27-06532-s001.zip › Section 6.1 plots and supplementary.pptx]

## Slide 1
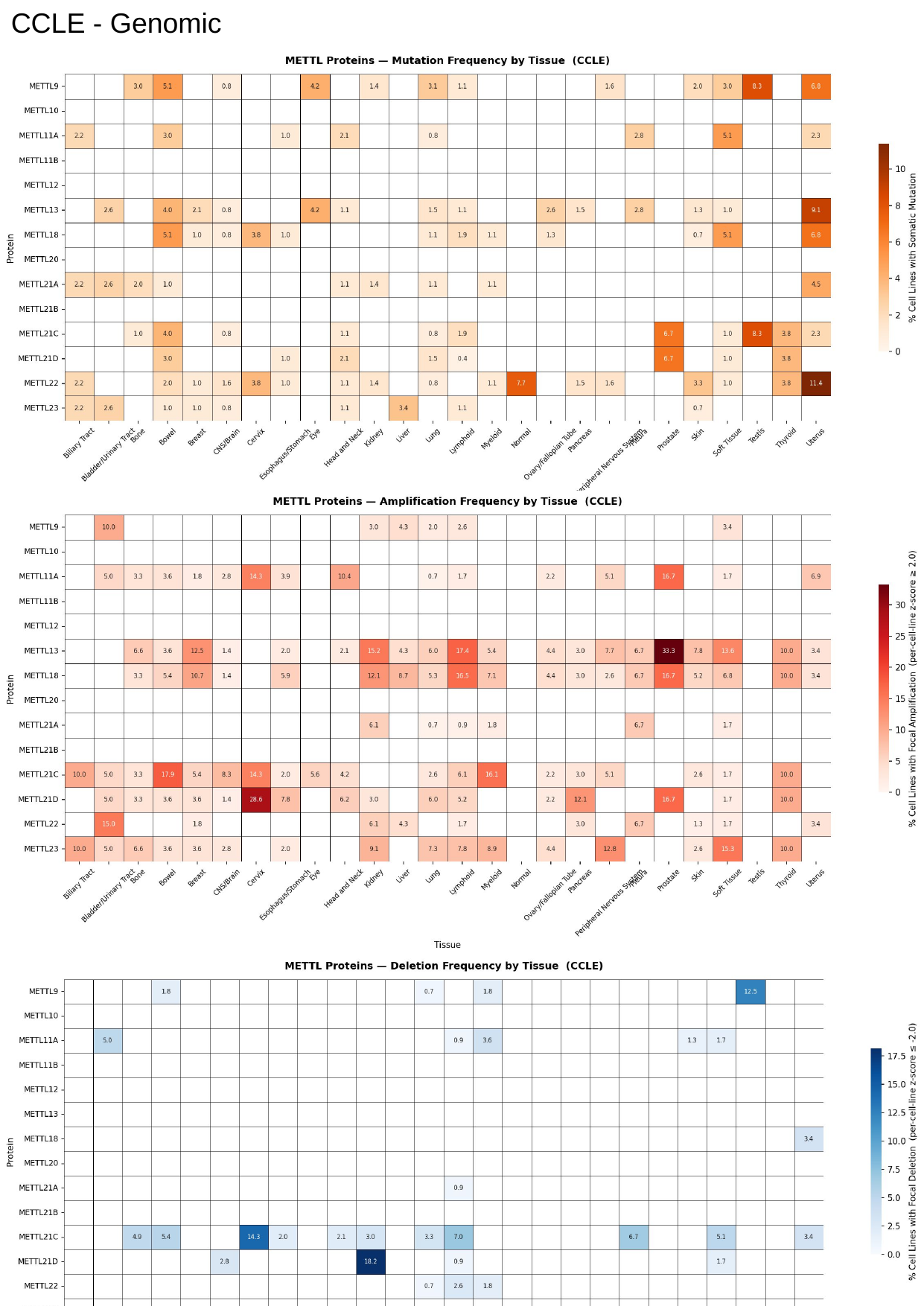

CCLE - Genomic

## Slide 2
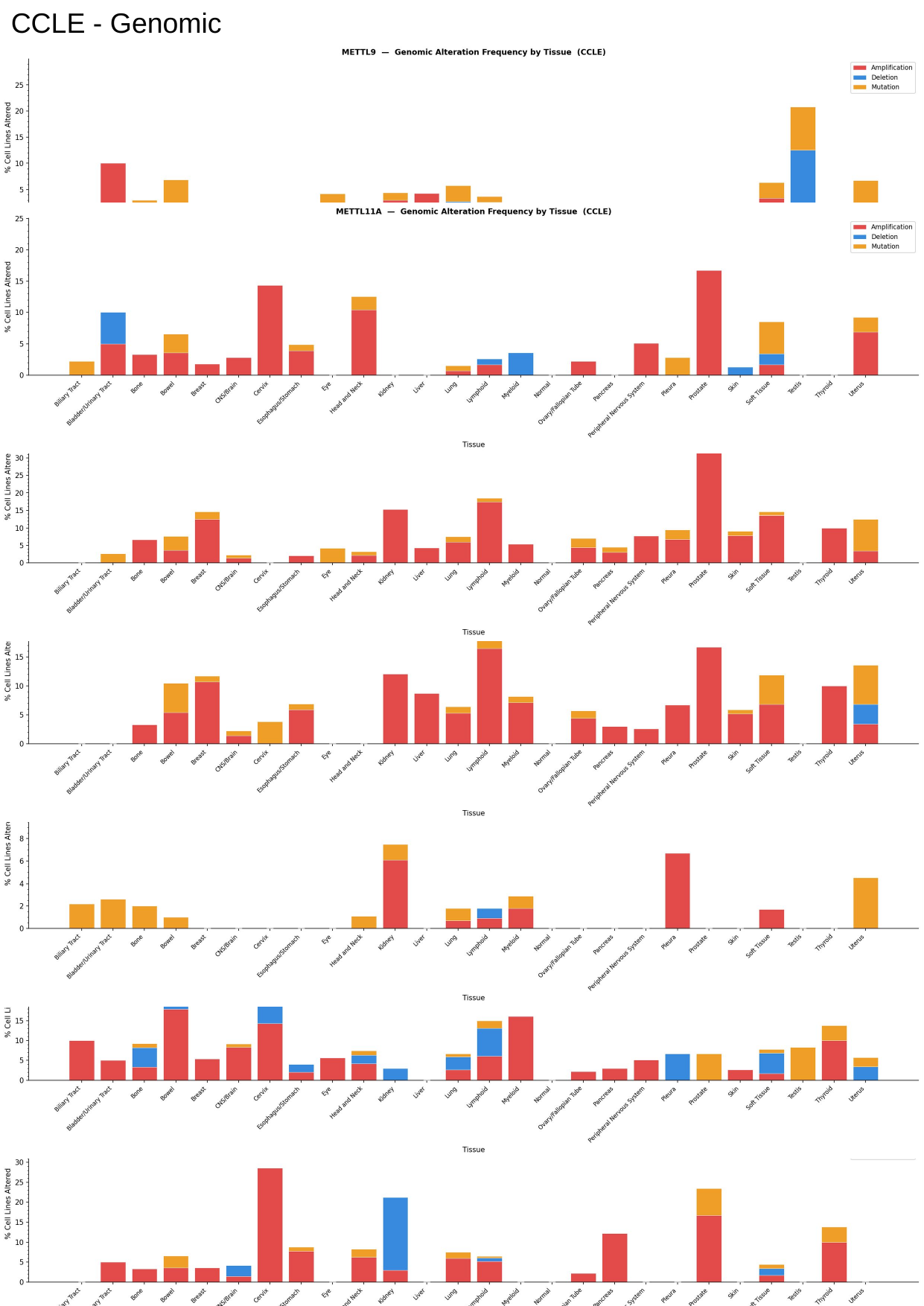

CCLE - Genomic

## Slide 3
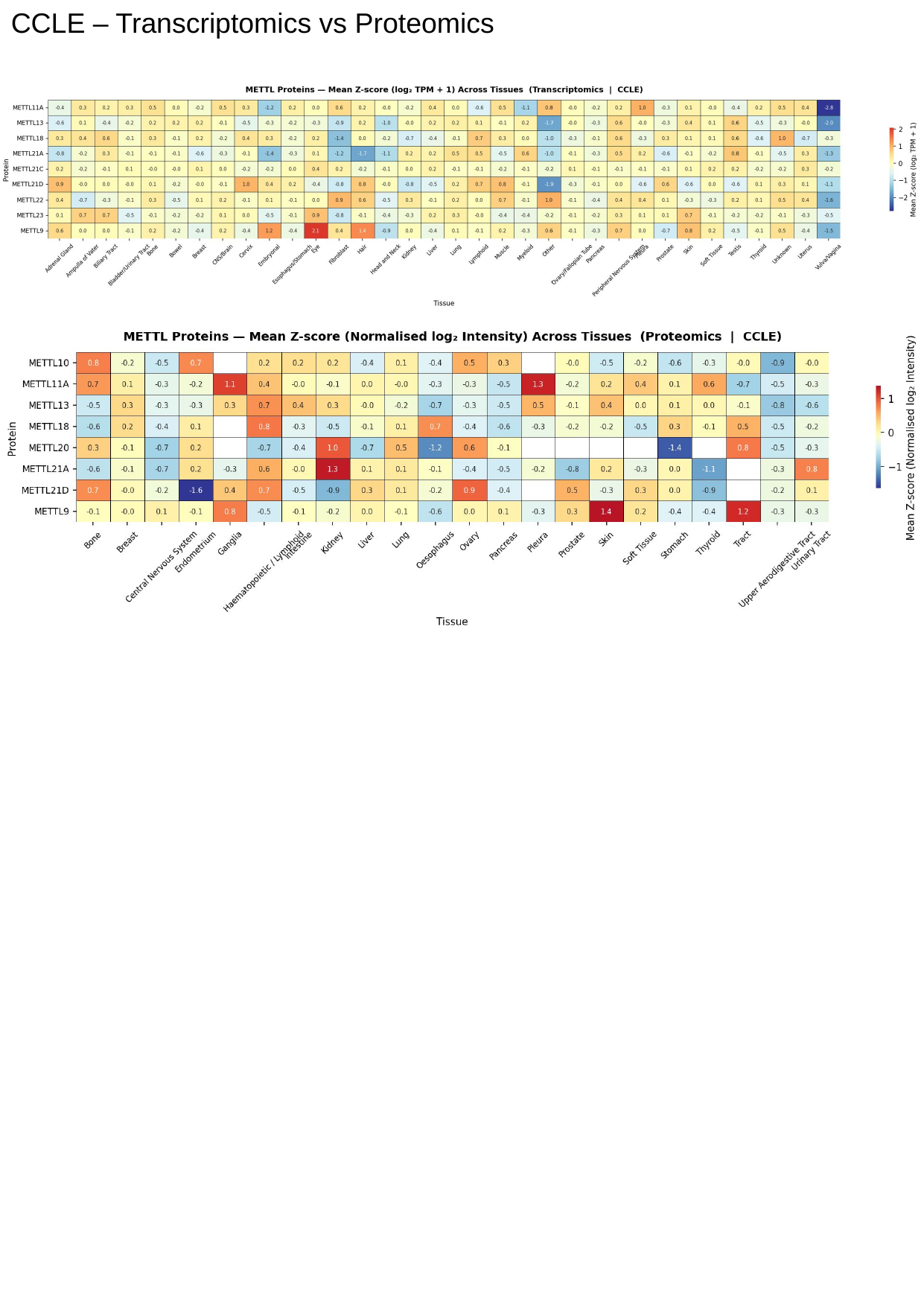

CCLE – Transcriptomics vs Proteomics

## Slide 4
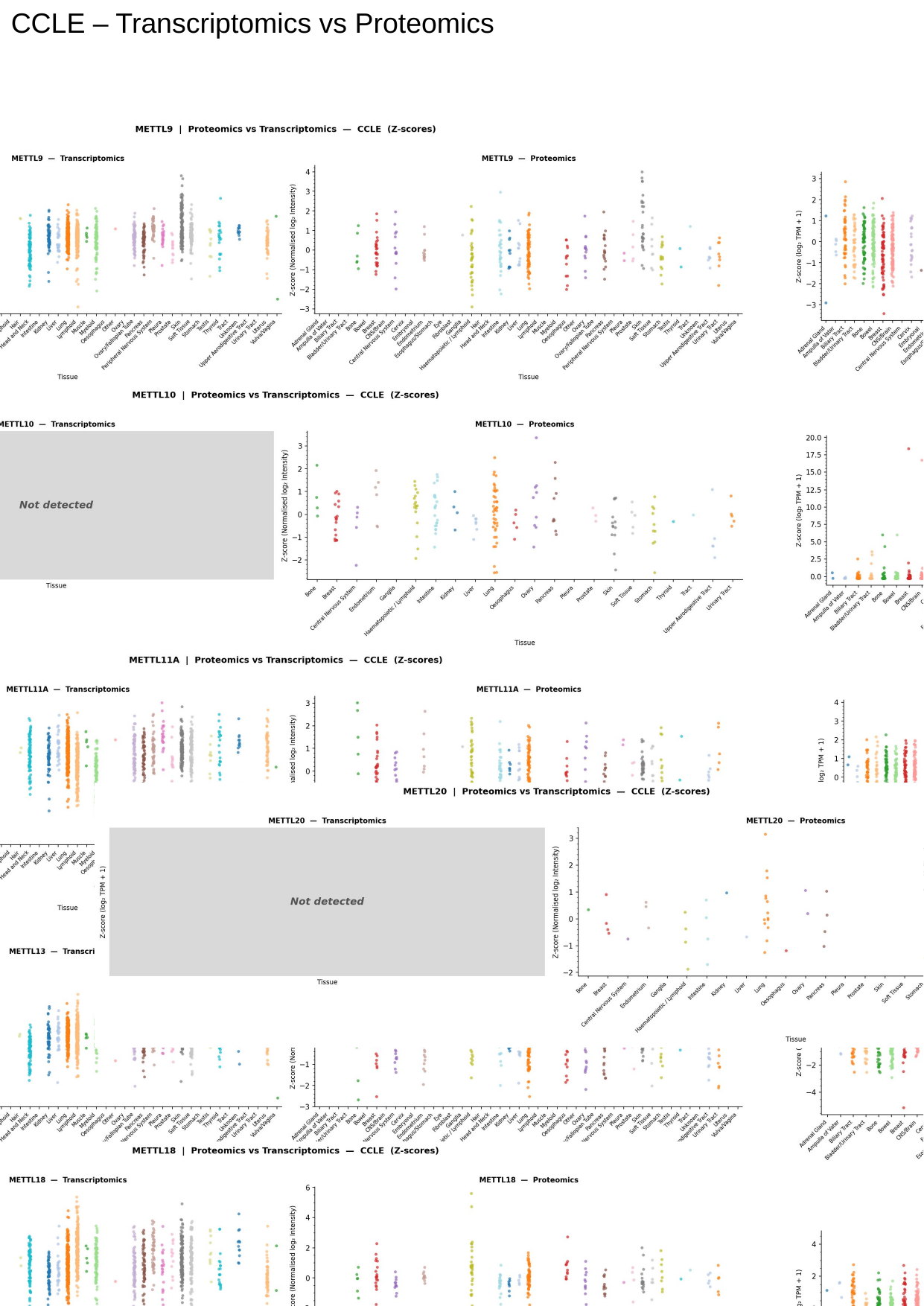

CCLE – Transcriptomics vs Proteomics

## Slide 5
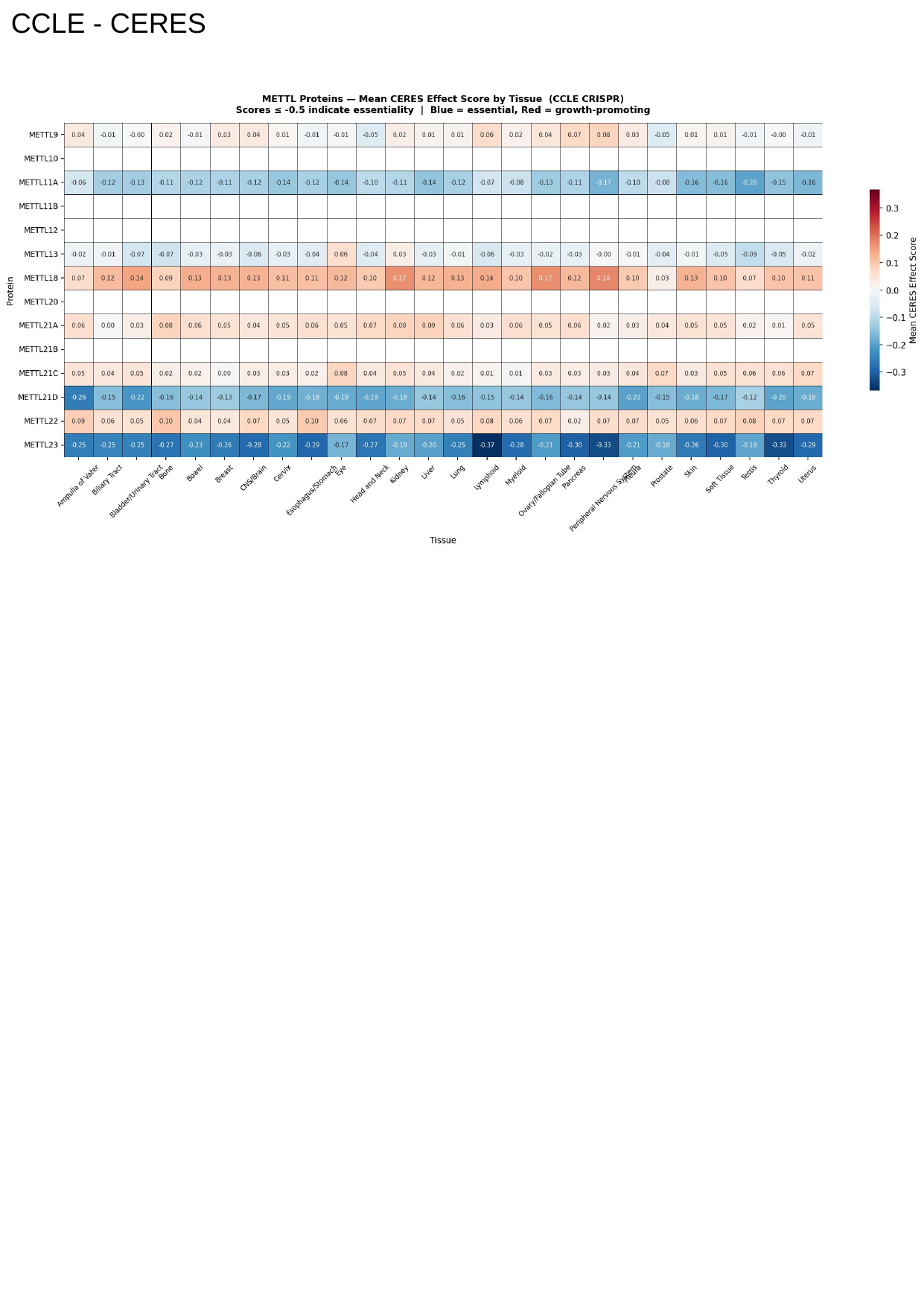

CCLE - CERES

## Slide 6
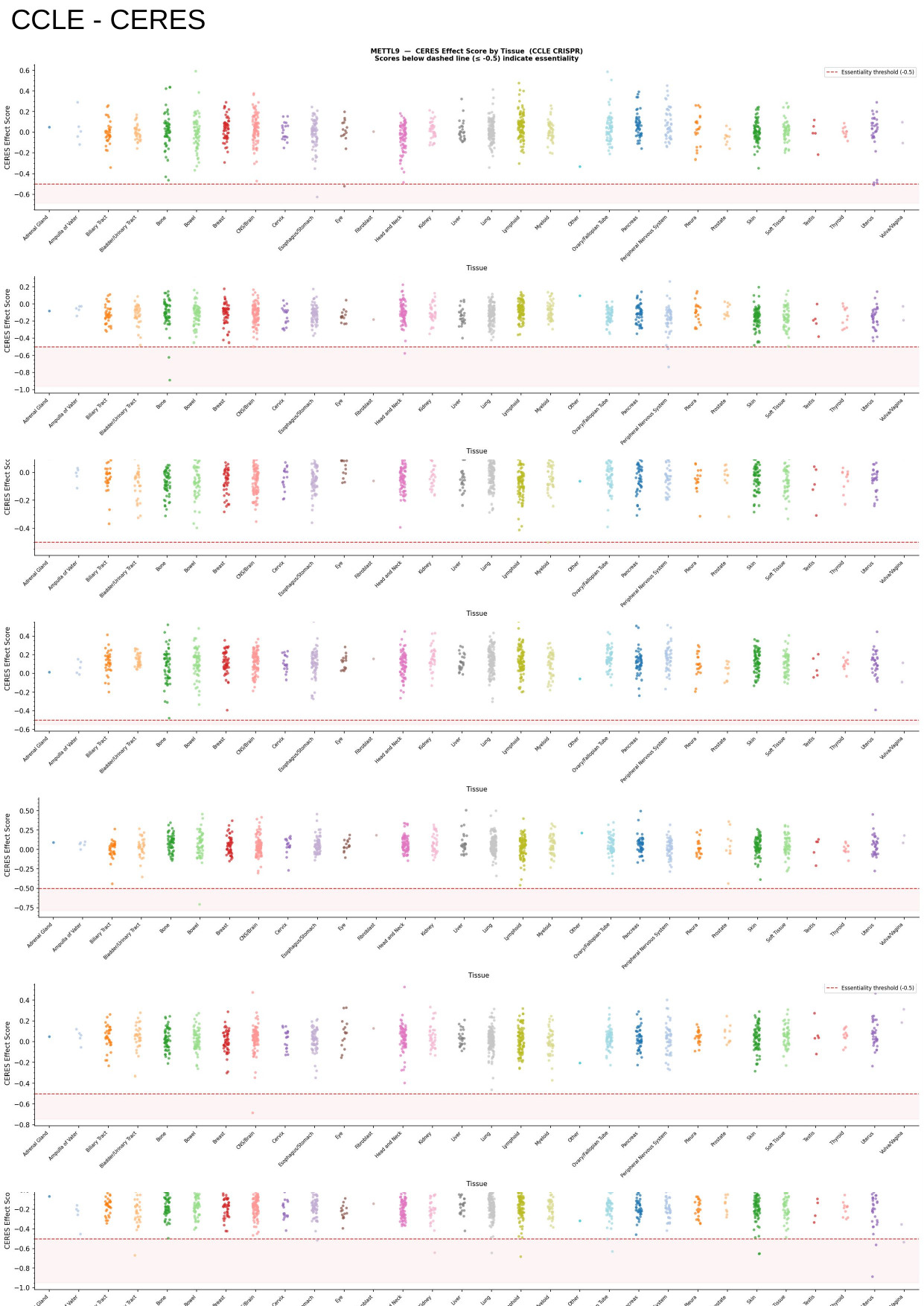

CCLE - CERES

## Slide 7
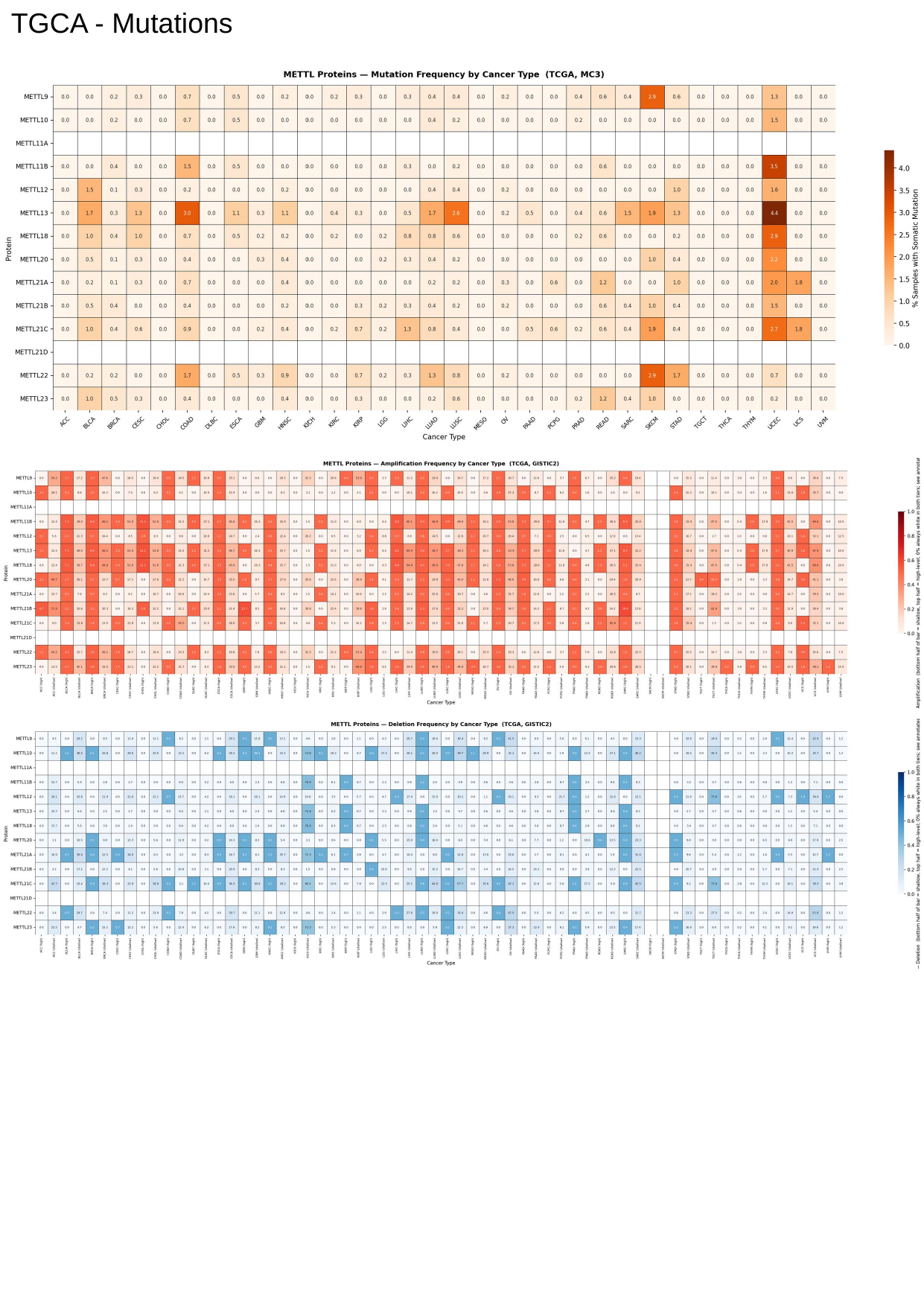

TGCA - Mutations

## Slide 8
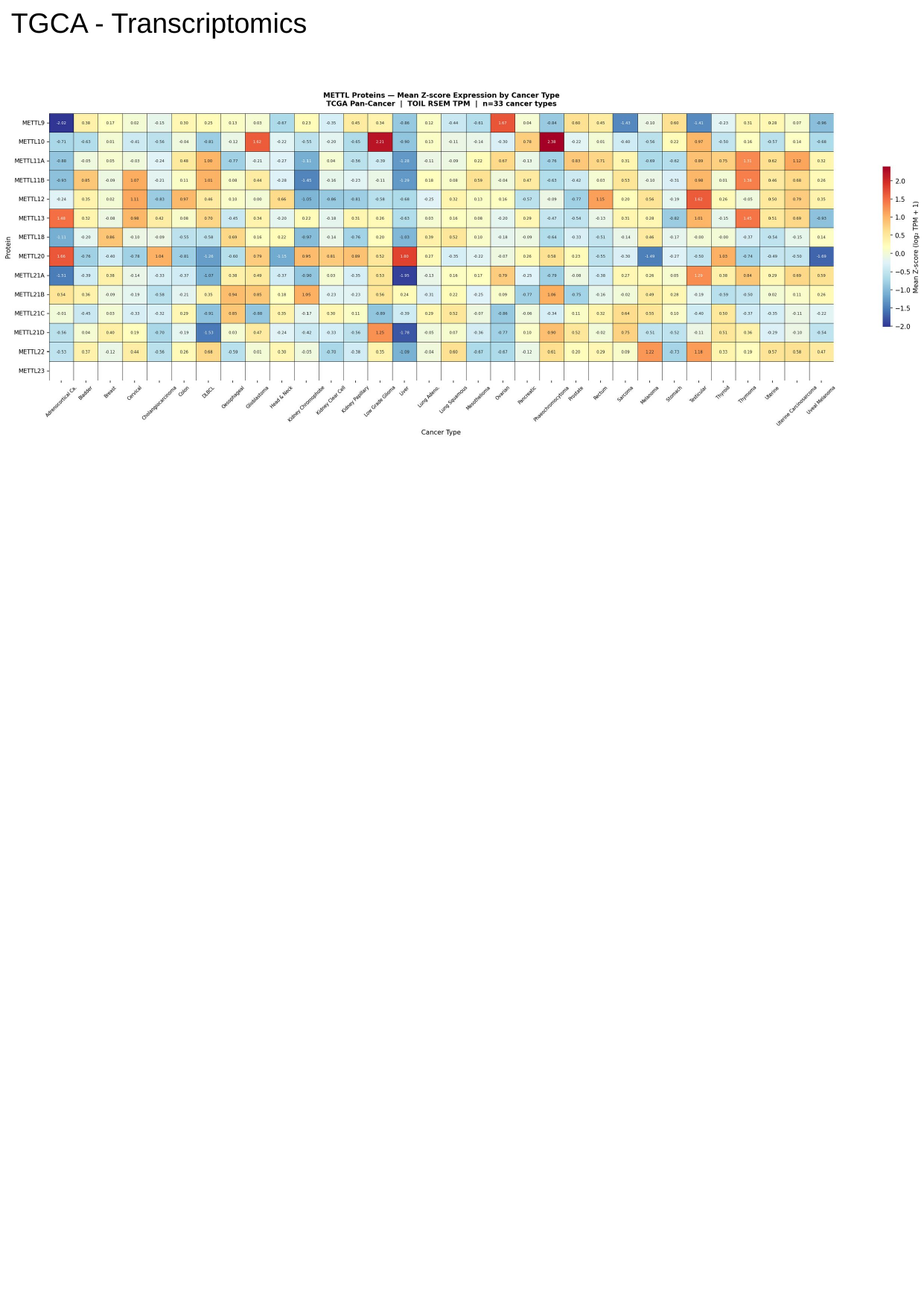

TGCA - Transcriptomics

## Slide 9
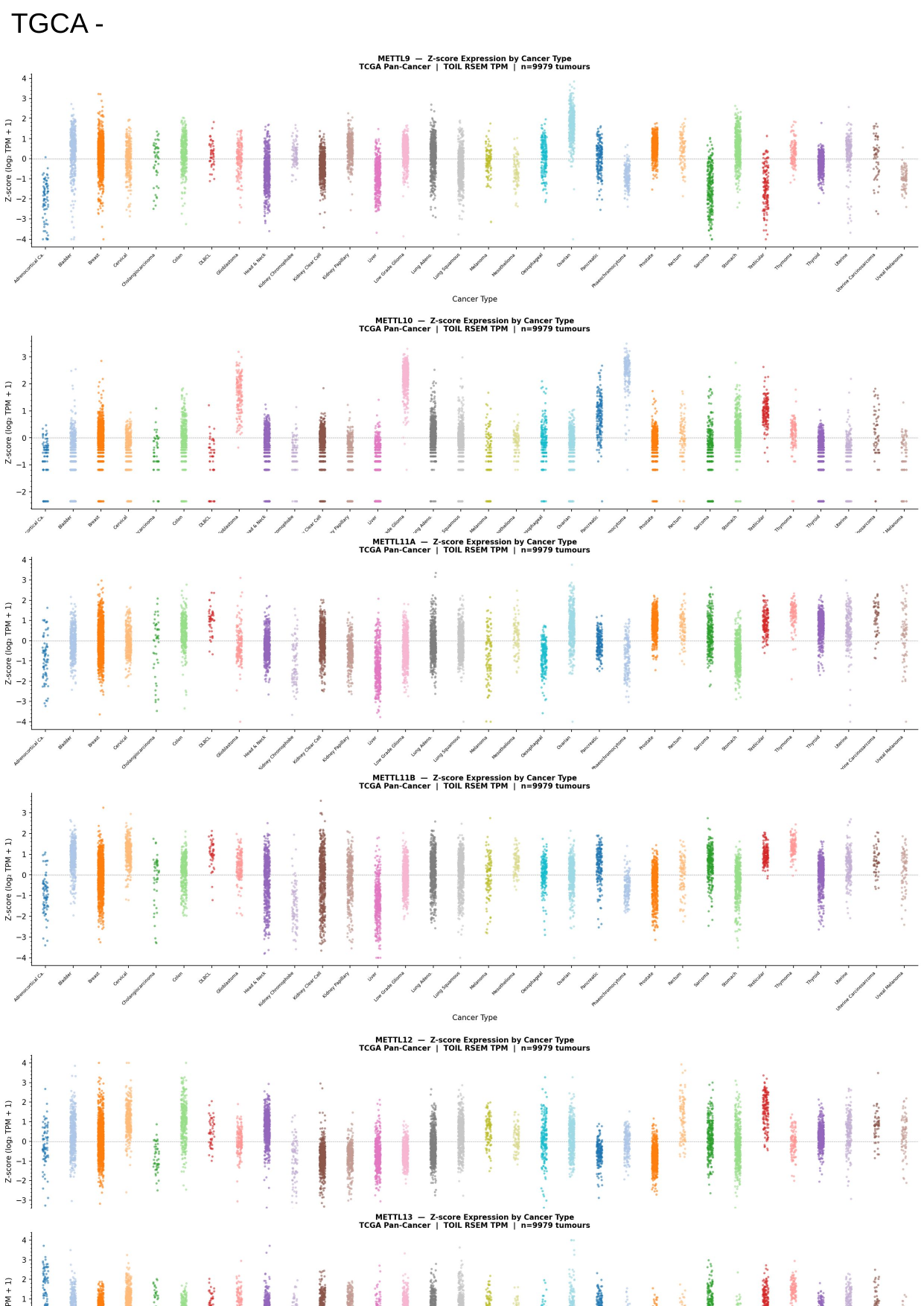

TGCA -

## Slide 10
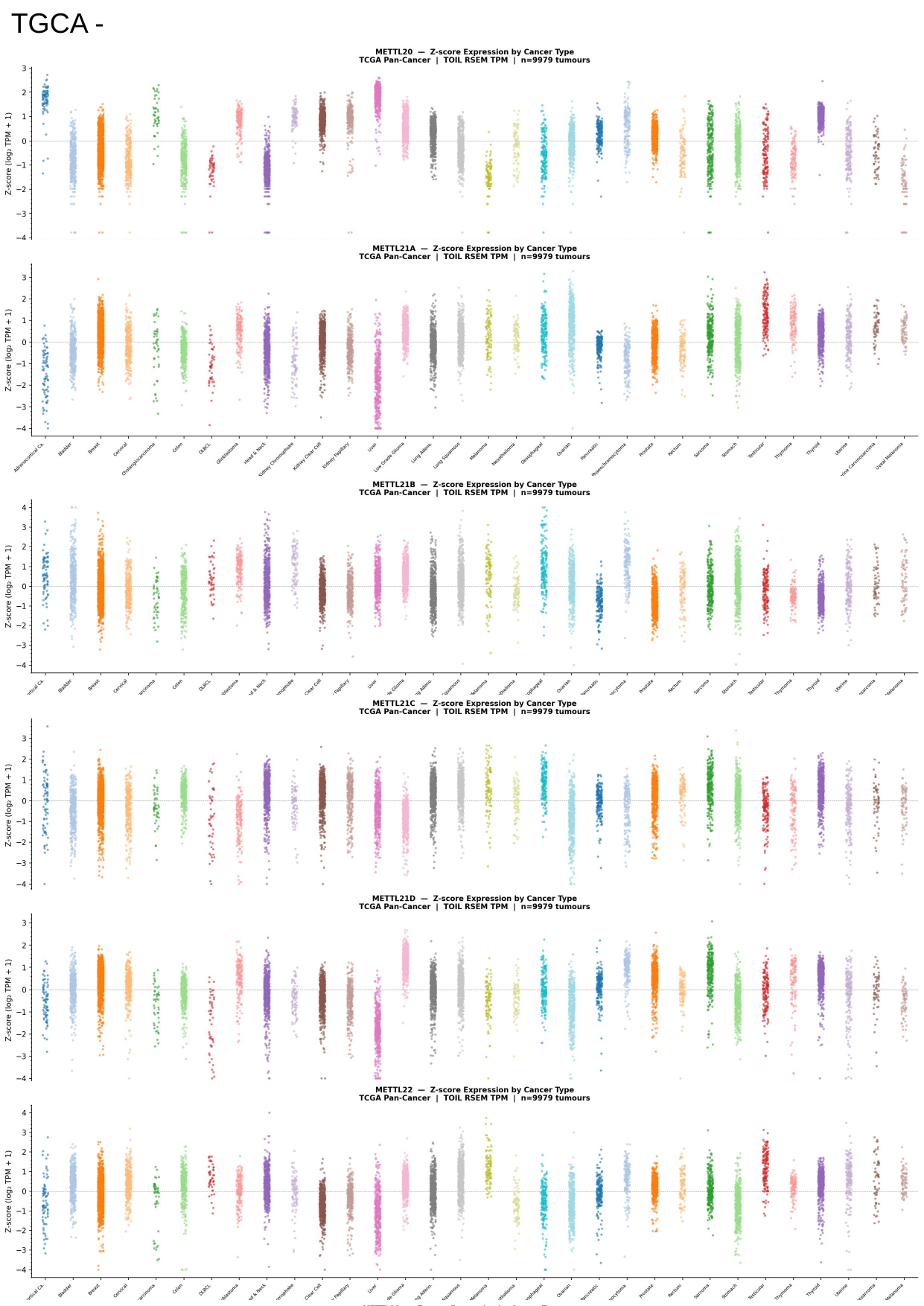

TGCA -

## Slide 11
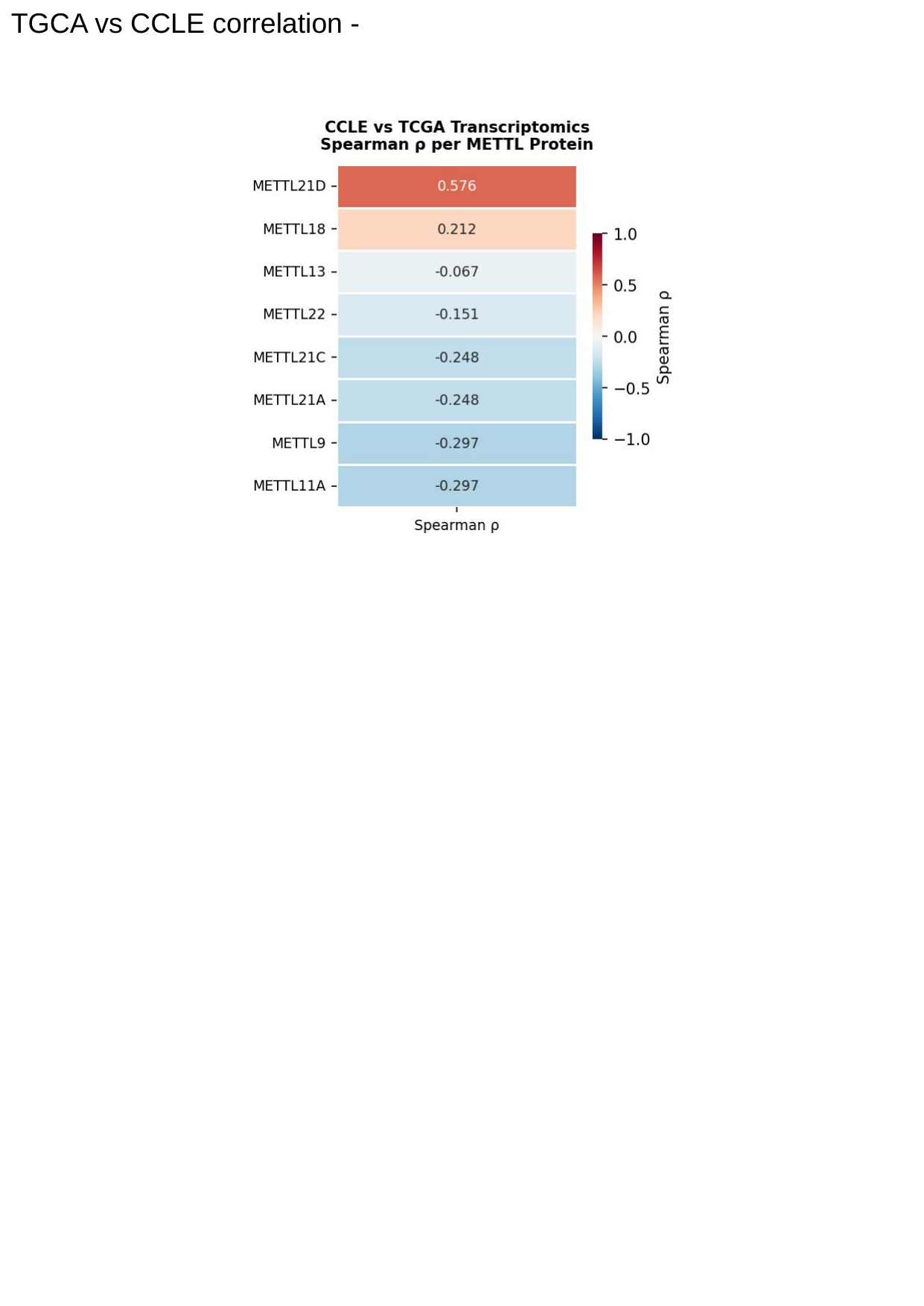

TGCA vs CCLE correlation -

## Slide 12
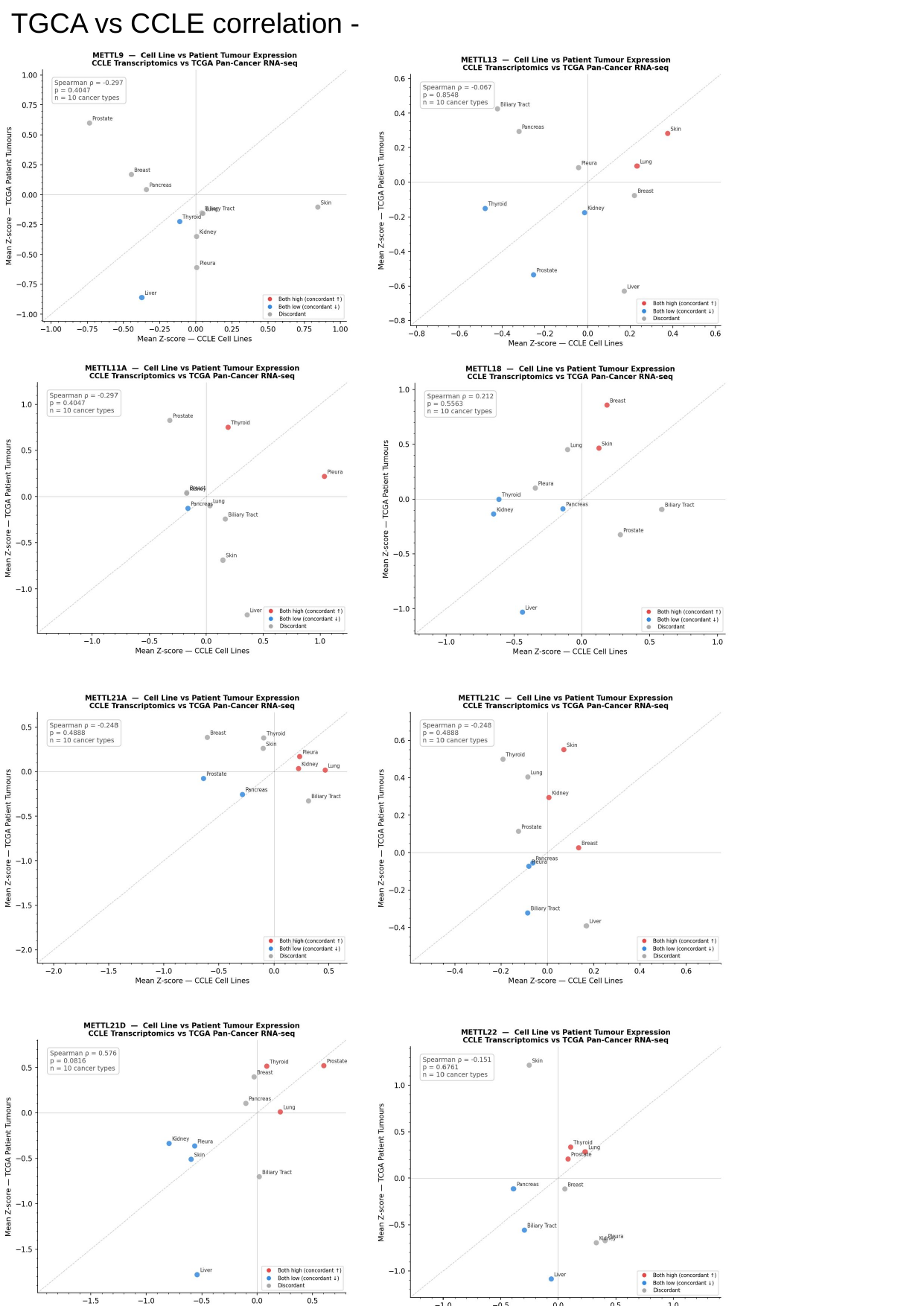

TGCA vs CCLE correlation -

## Slide 13
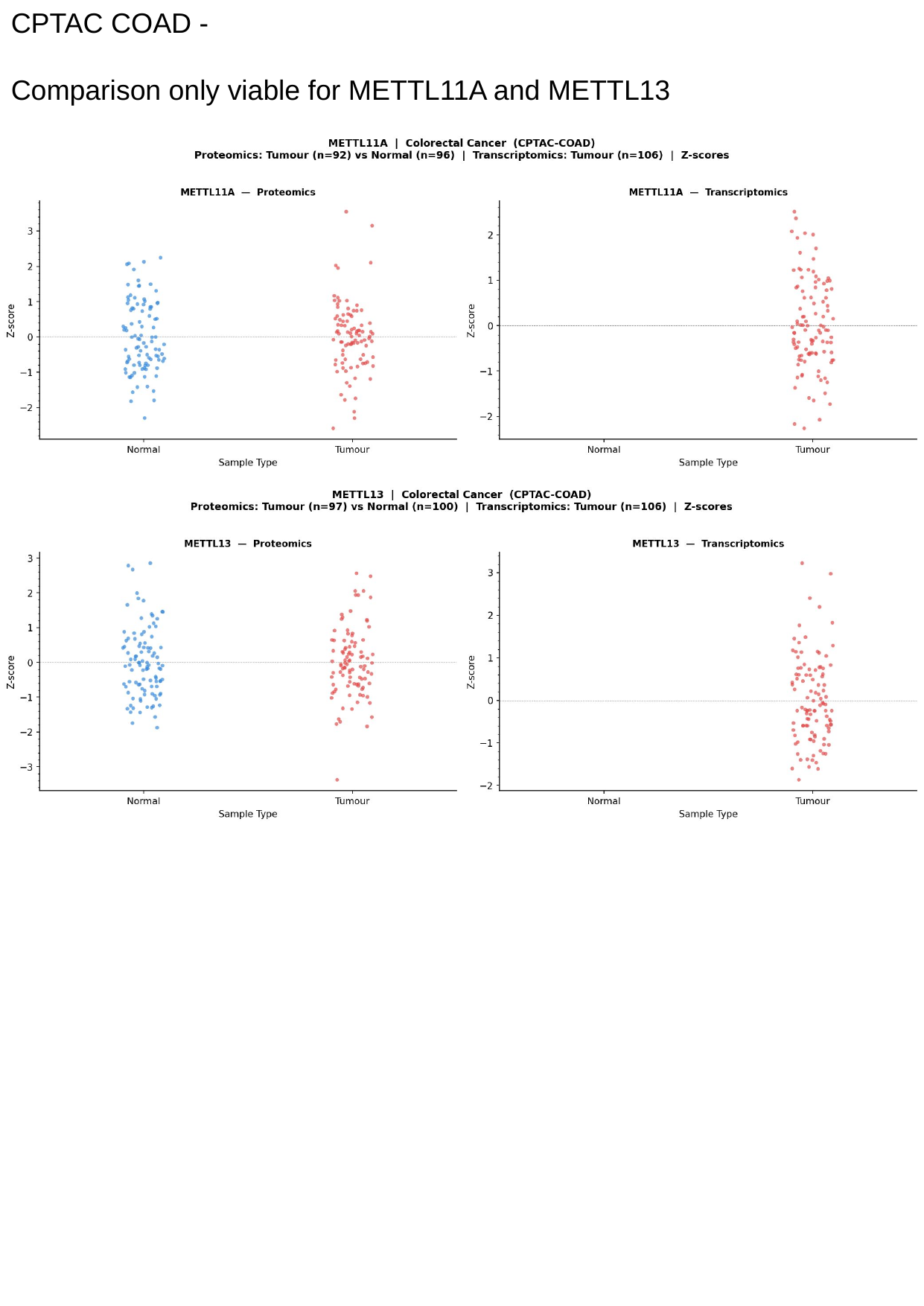

CPTAC COAD -
Comparison only viable for METTL11A and METTL13

## Slide 14
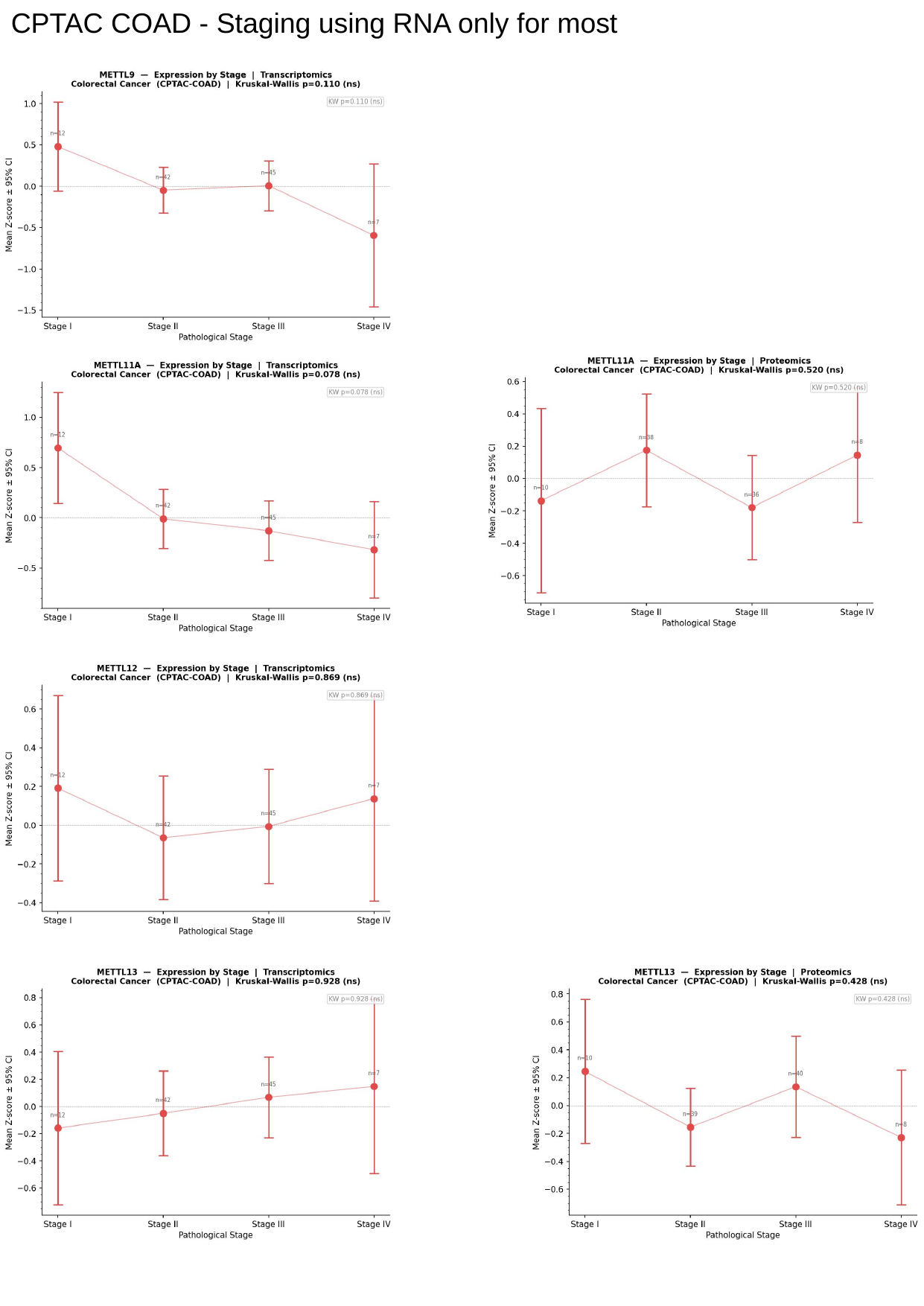

CPTAC COAD - Staging using RNA only for most

## Slide 15
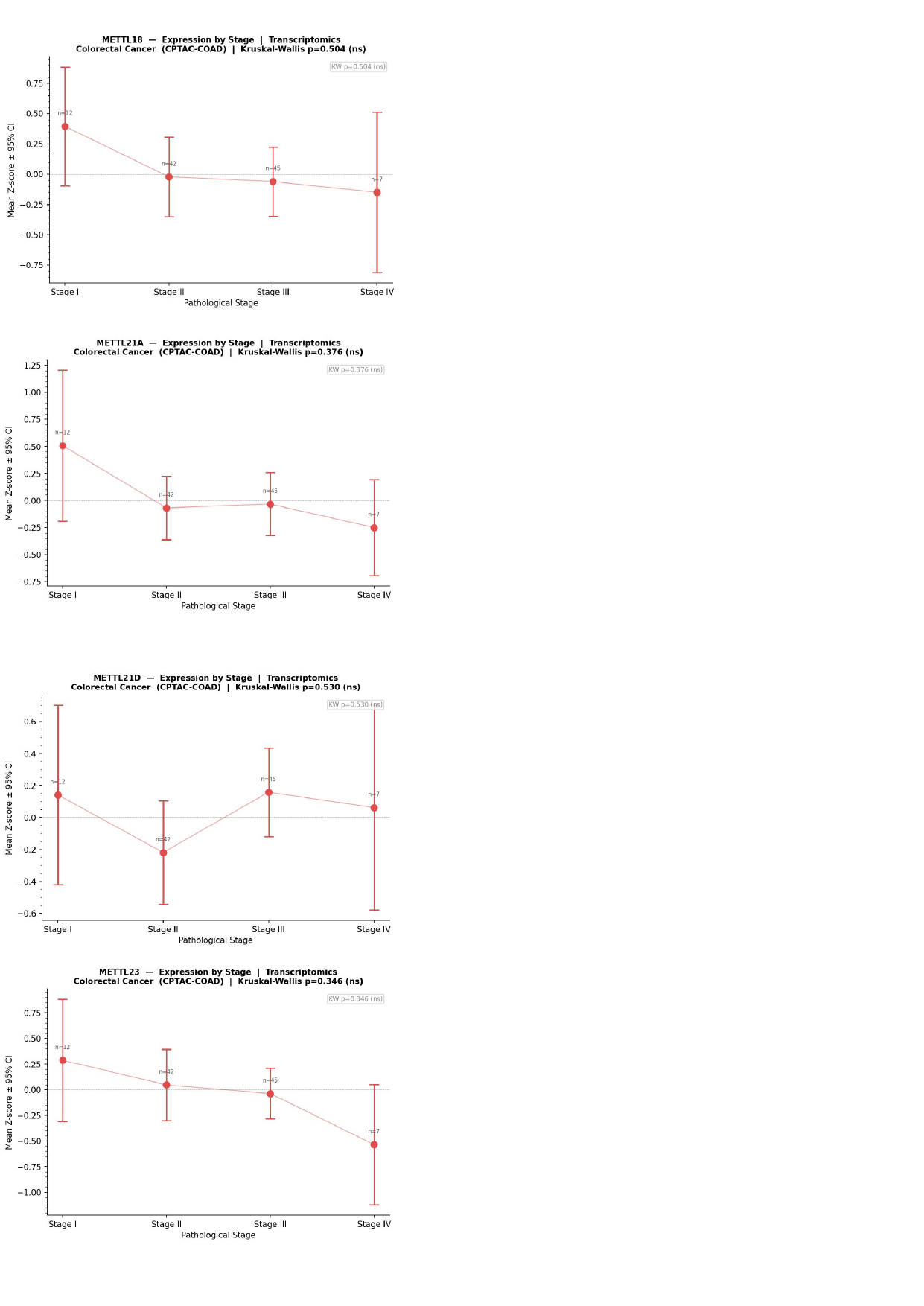

## Slide 16
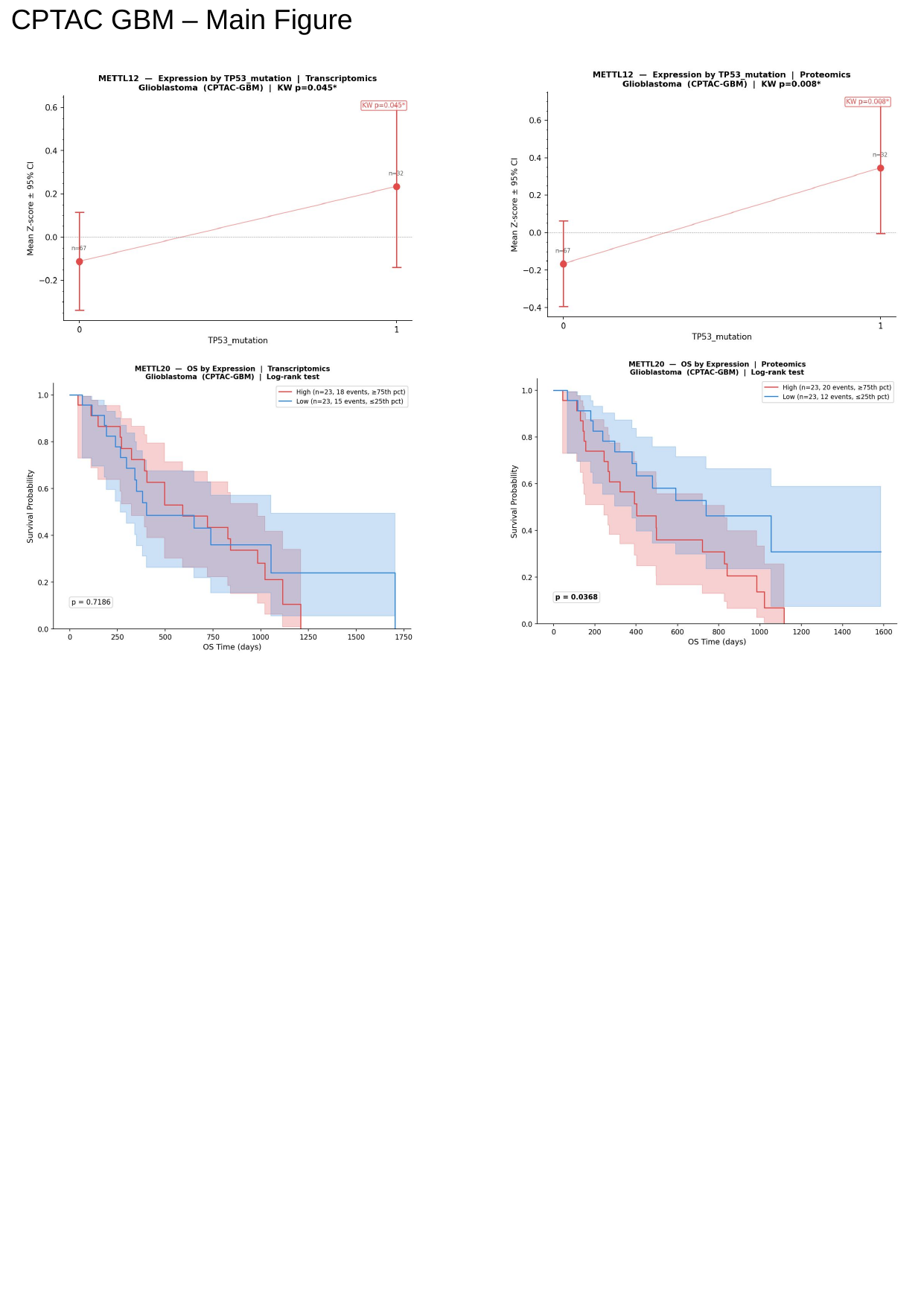

CPTAC GBM – Main Figure

## Slide 17
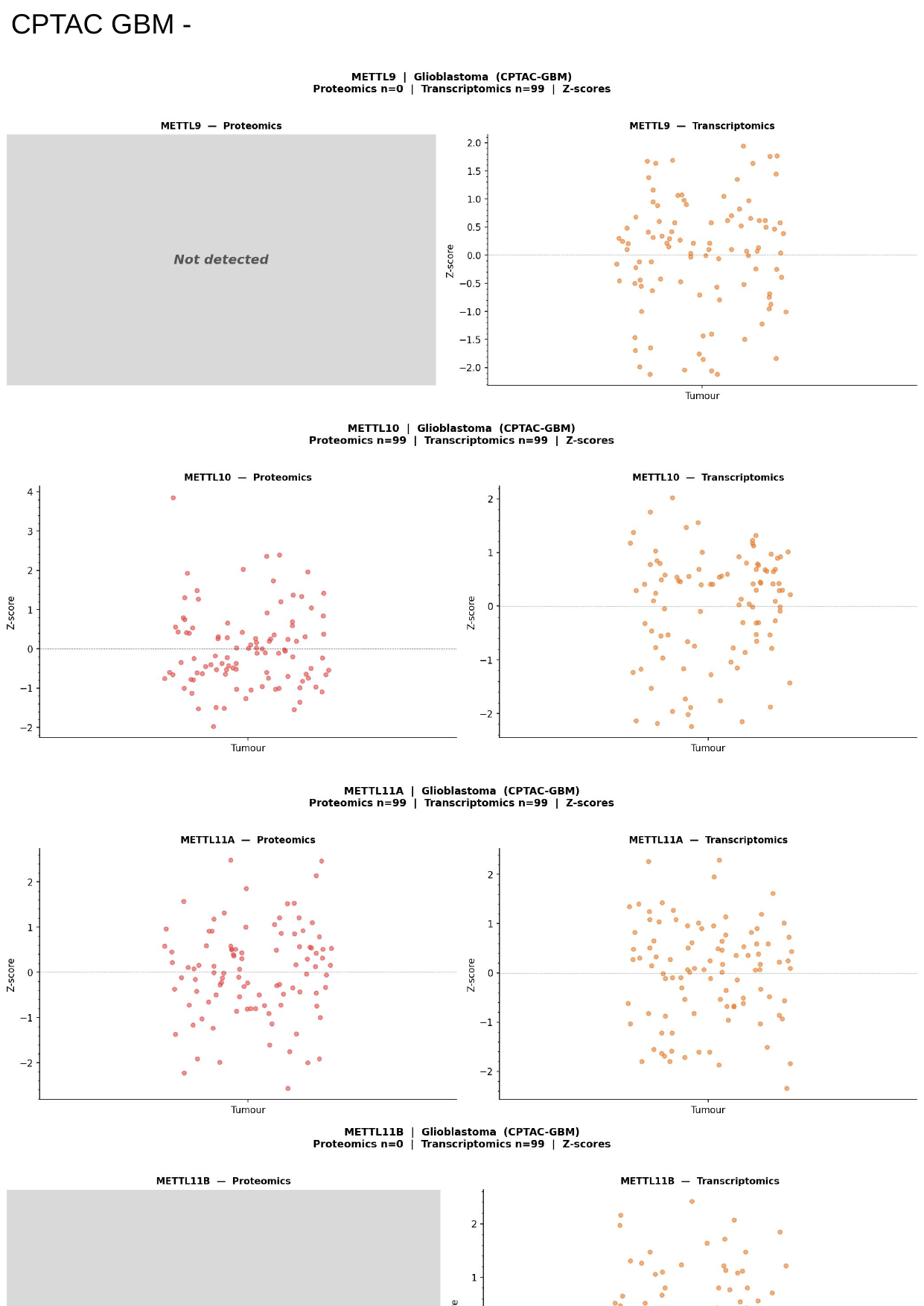

CPTAC GBM -
Comparison

## Slide 18
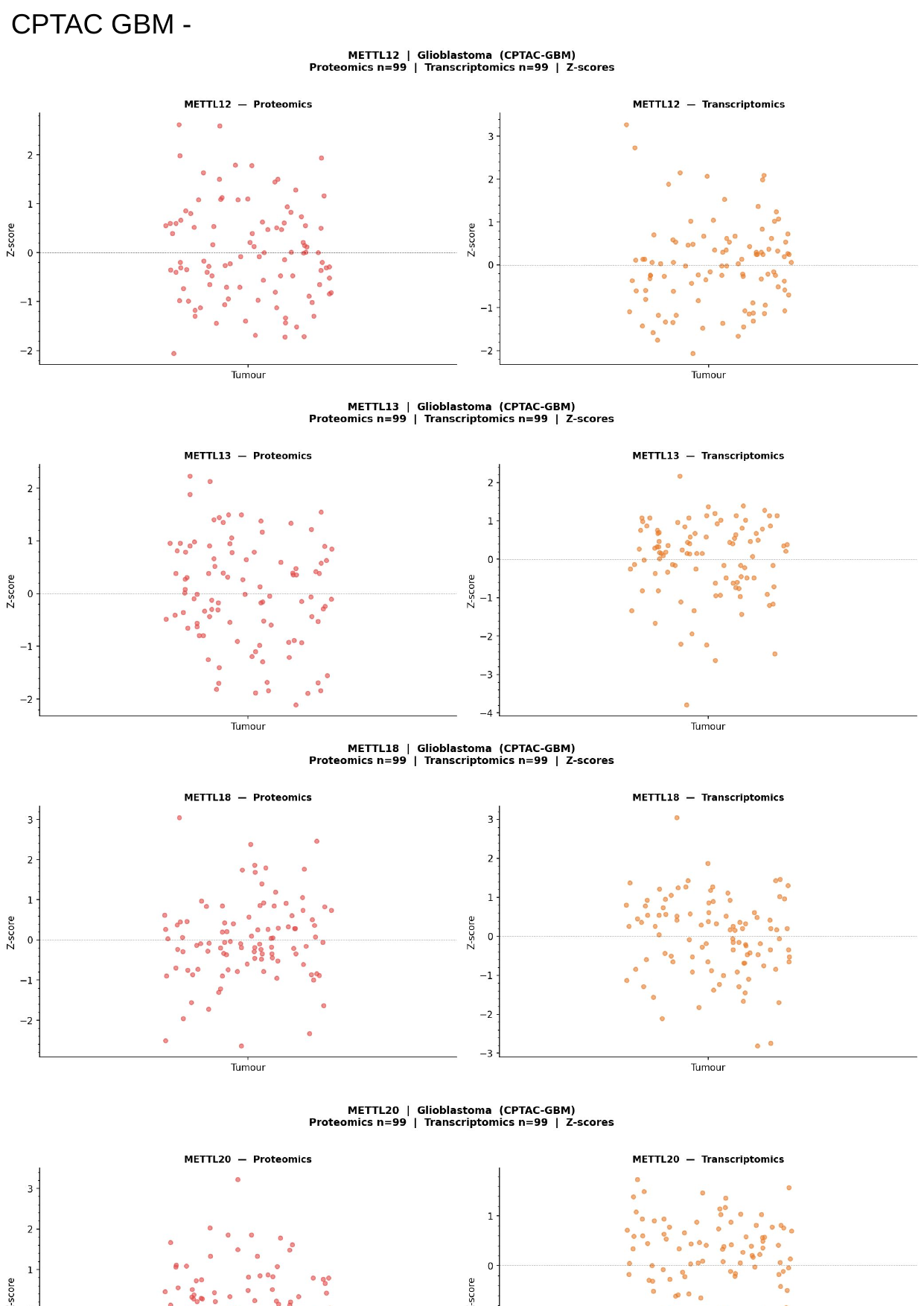

CPTAC GBM -

## Slide 19
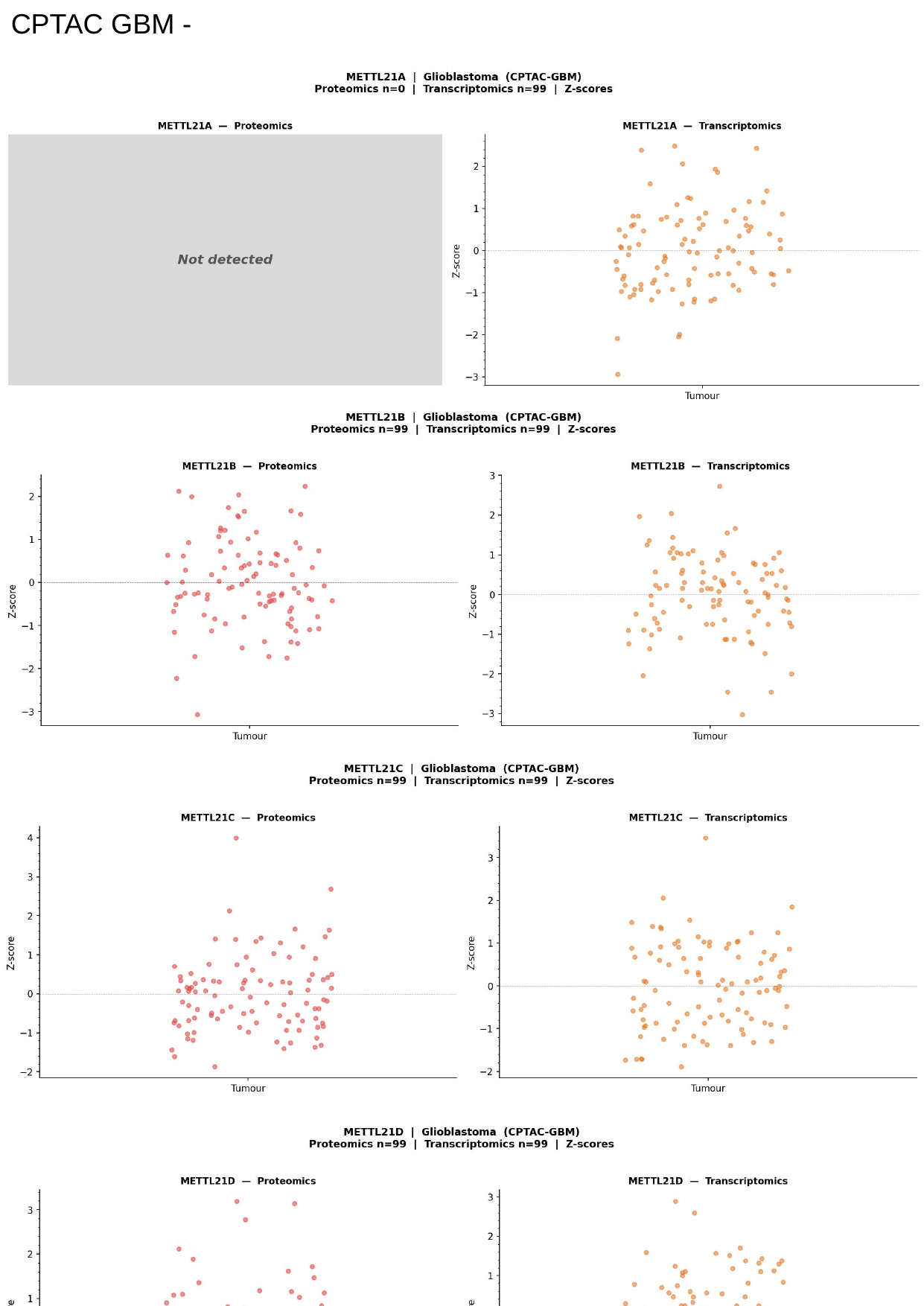

CPTAC GBM -

## Slide 20
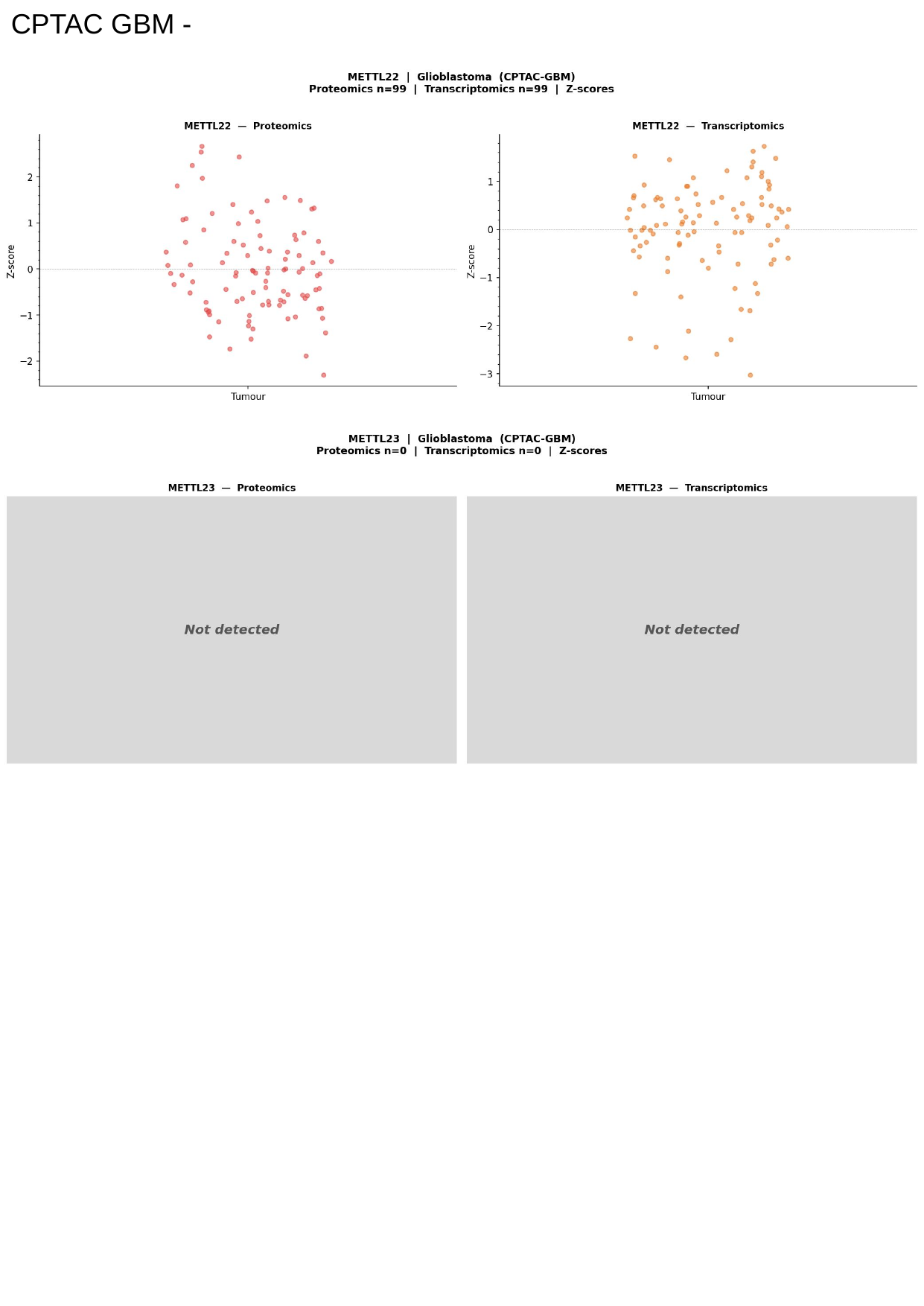

CPTAC GBM -

## Slide 21
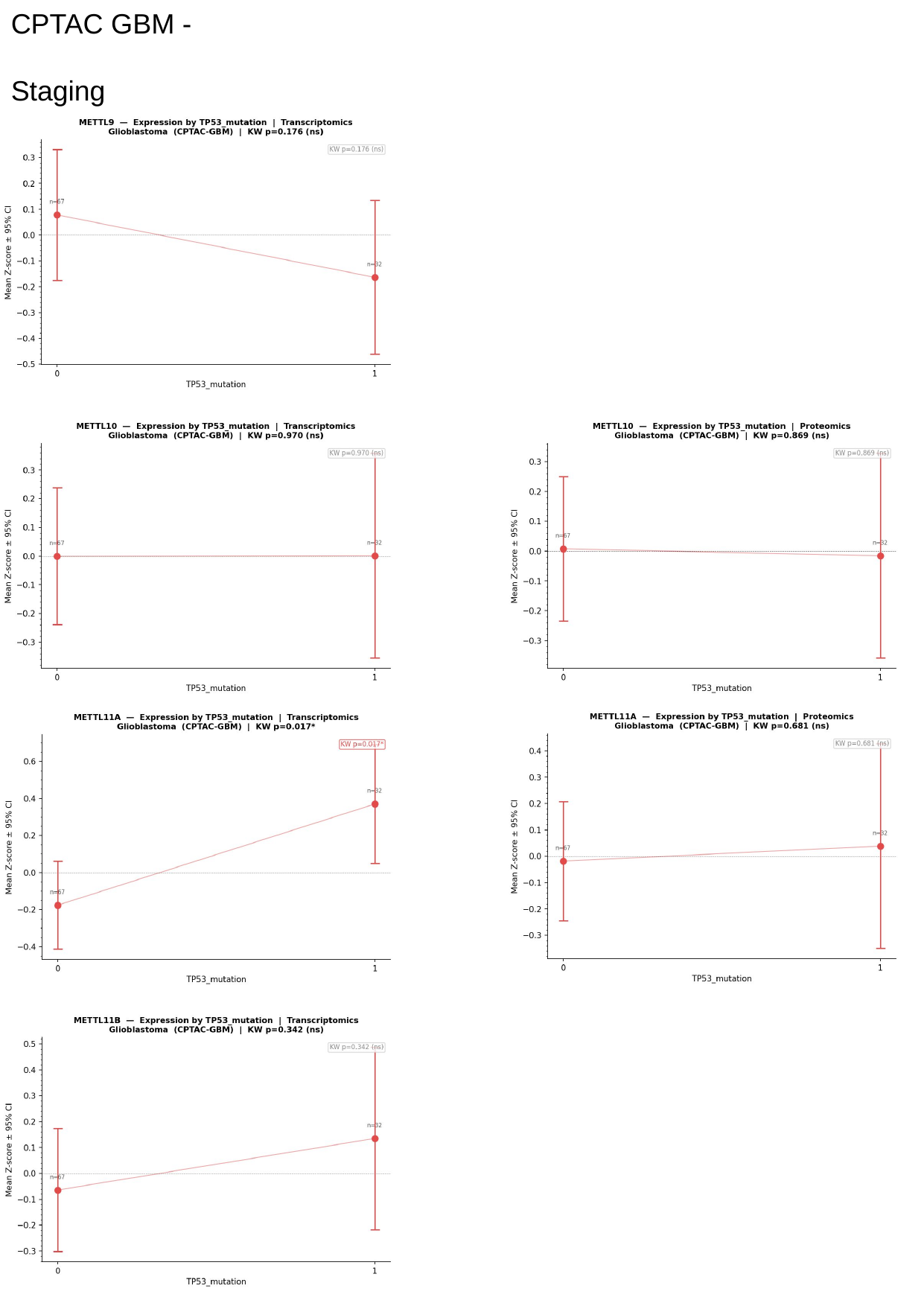

CPTAC GBM -
Staging

## Slide 22
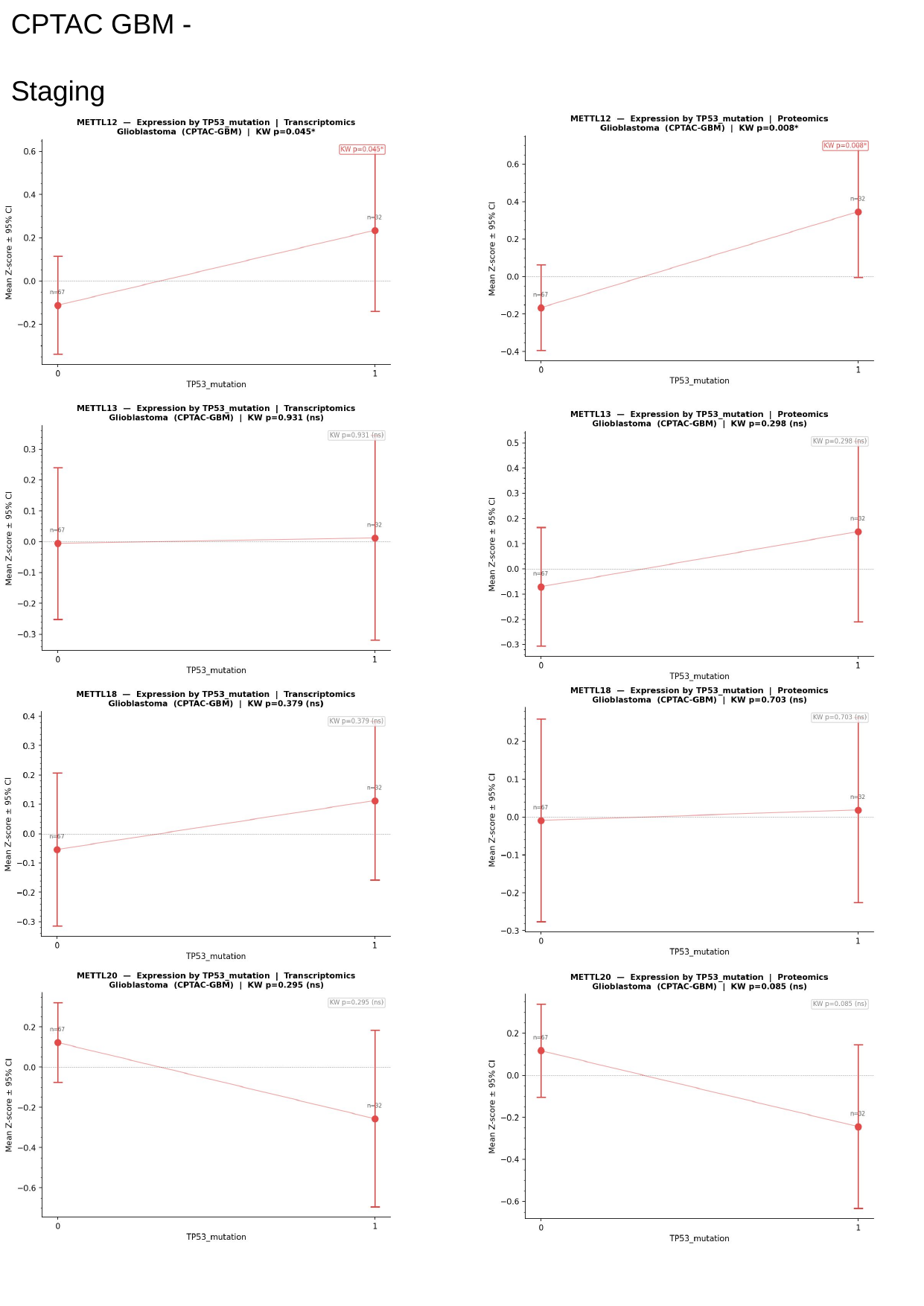

CPTAC GBM -
Staging

## Slide 23
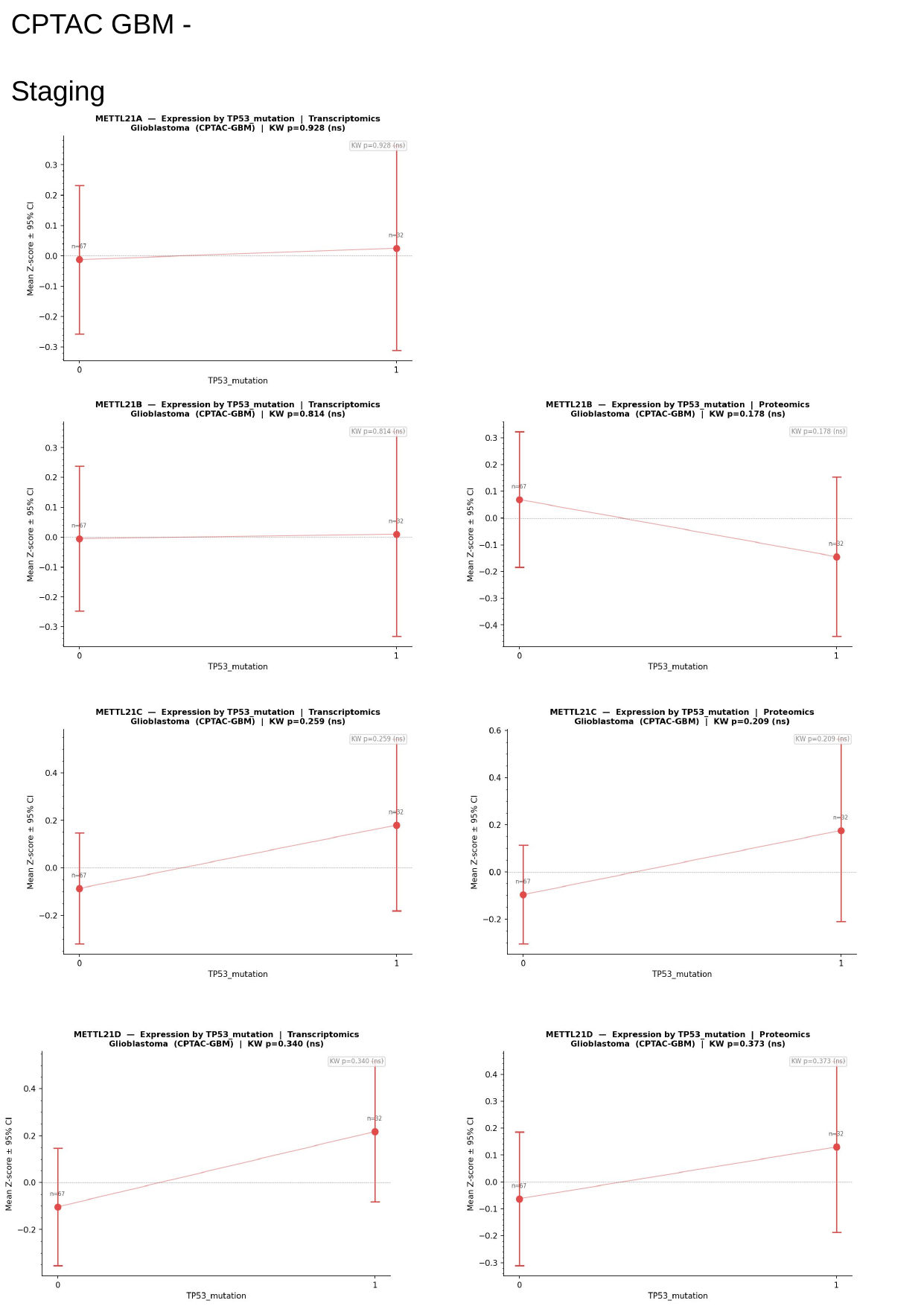

CPTAC GBM -
Staging

## Slide 24
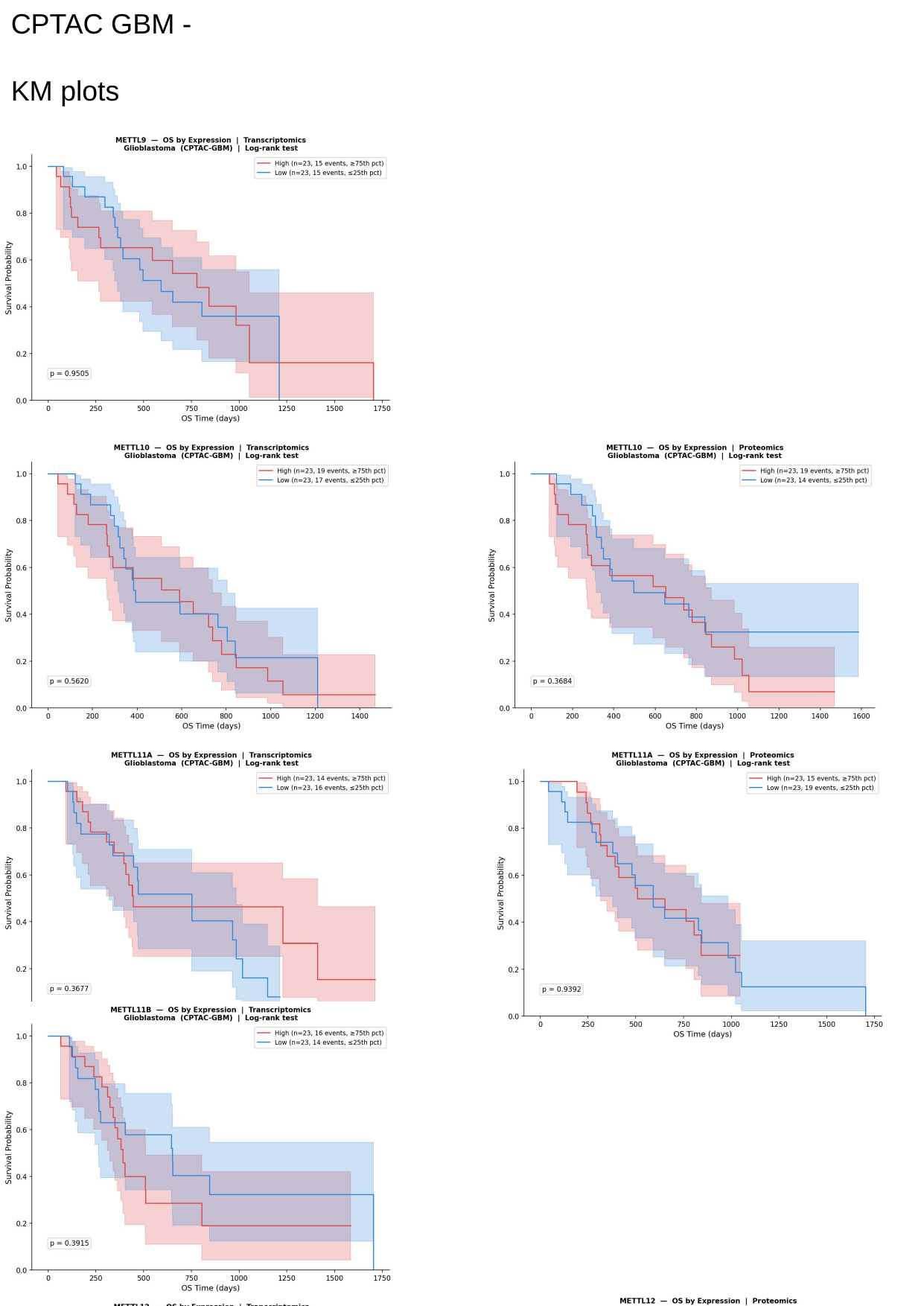

CPTAC GBM -
KM plots

## Slide 25
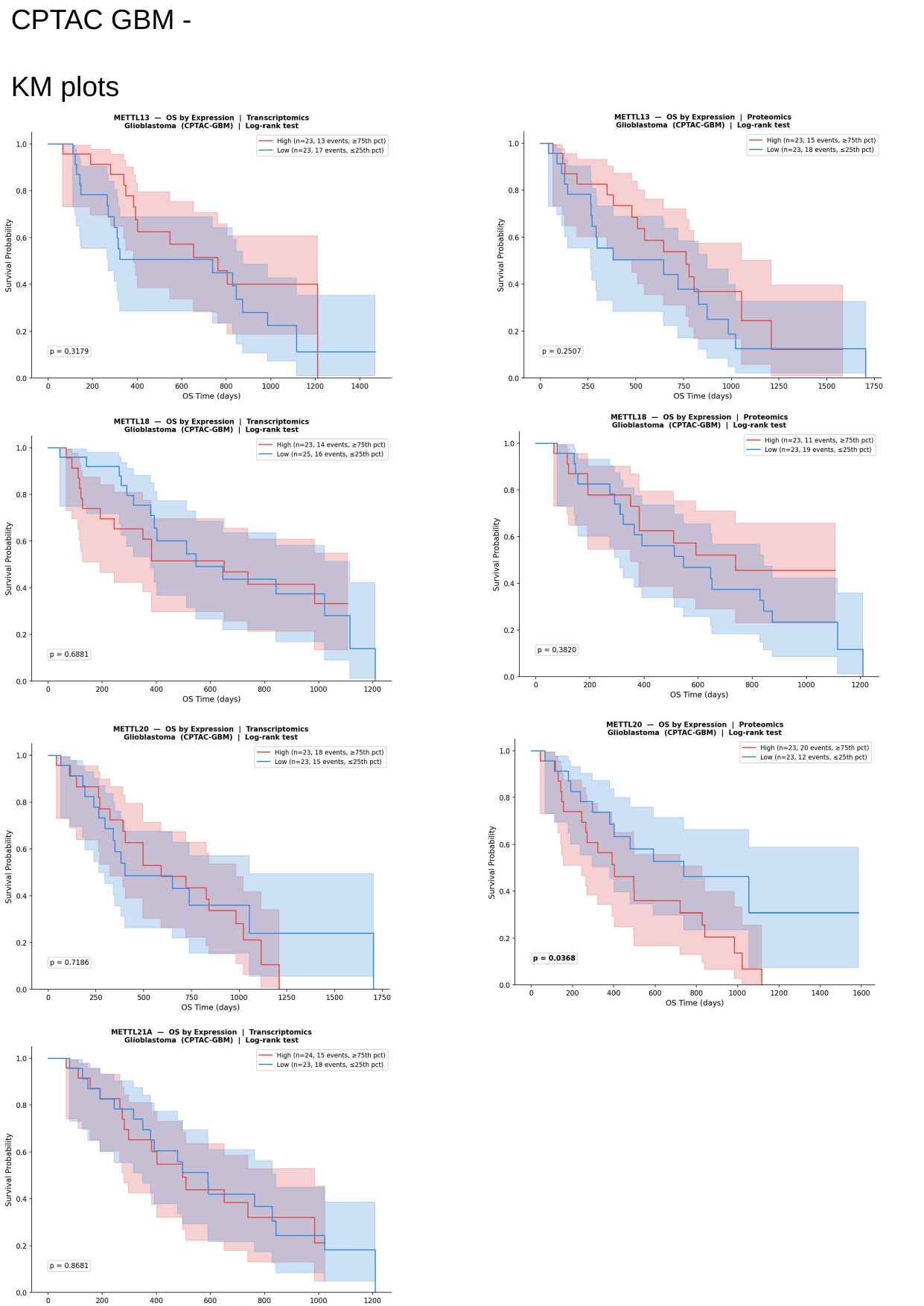

CPTAC GBM -
KM plots

## Slide 26
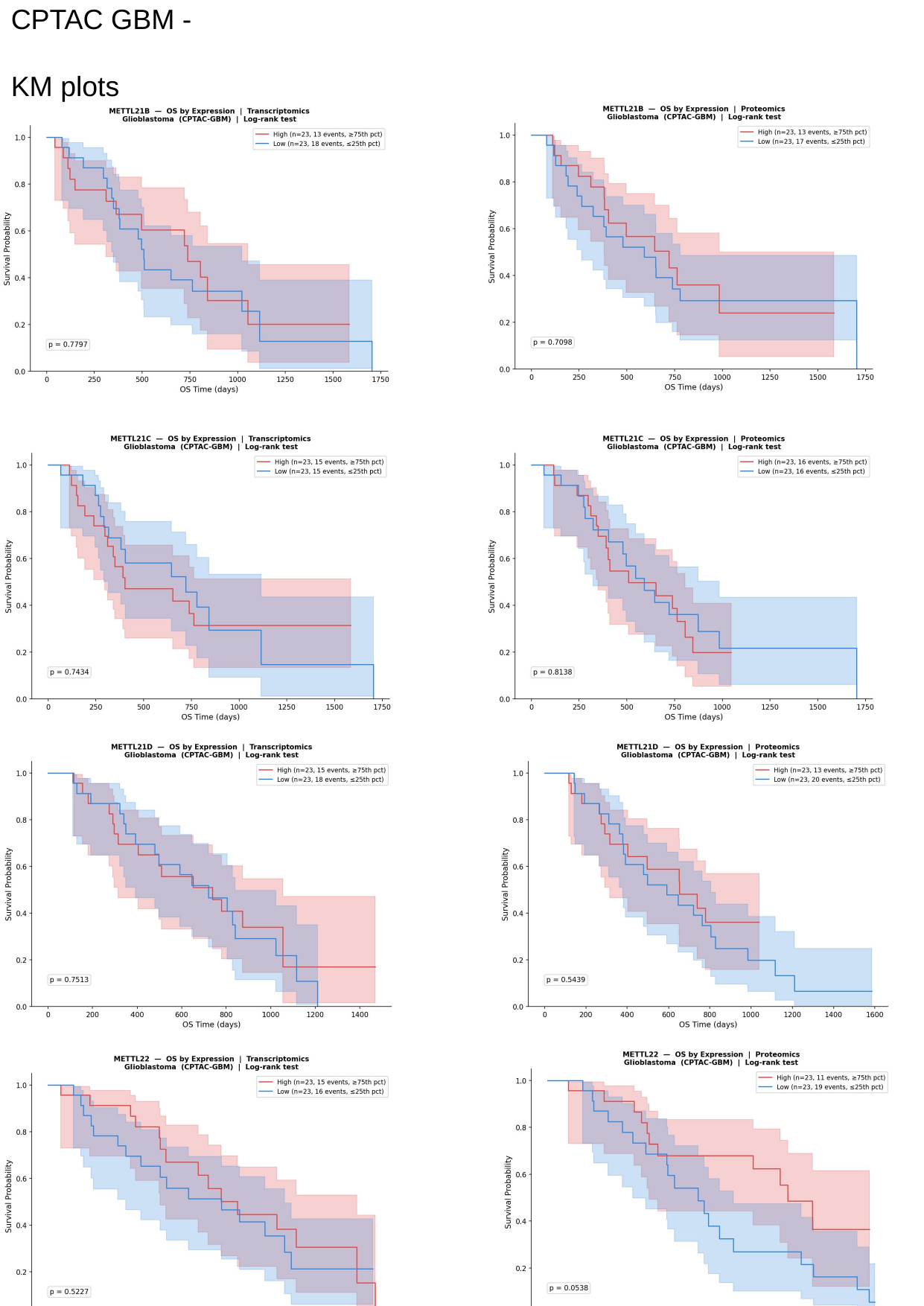

CPTAC GBM -
KM plots
